# The Influence of CYP2D6 and CYP2C19 Genetic Variation on Diabetes Mellitus Risk in People Taking Antidepressants and Antipsychotics

**DOI:** 10.3390/genes12111758

**Published:** 2021-11-03

**Authors:** Isabelle Austin-Zimmerman, Marta Wronska, Baihan Wang, Haritz Irizar, Johan H. Thygesen, Anjali Bhat, Spiros Denaxas, Ghazaleh Fatemifar, Chris Finan, Jasmine Harju-Seppänen, Olga Giannakopoulou, Karoline Kuchenbaecker, Eirini Zartaloudi, Andrew McQuillin, Elvira Bramon

**Affiliations:** 1 Department of Mental Health Neurosciences, Division of Psychiatry, University College London, London W1T 7BN, UK; marta.wronska.19@ucl.ac.uk (M.W.); wang.18@ucl.ac.uk (B.W.); a.irizar@ucl.ac.uk (H.I.); j.thygesen@ucl.ac.uk (J.H.T.); anjali.bhat.14@ucl.ac.uk (A.B.); jasmine.harju-seppanen@ucl.ac.uk (J.H.-S.); o.giannakopoulou@ucl.ac.uk (O.G.); k.kuchenbaecker@ucl.ac.uk (K.K.); e.zartaloudi@ucl.ac.uk (E.Z.); a.mcquillin@ucl.ac.uk (A.M.); 2 Social, Genetic and Developmental Psychiatry Centre, Institute of Psychiatry, Psychology and Neuroscience, King’s College London, London SE5 8AF, UK; 3 Department of Genetics & Genomic Sciences, Icahn School of Medicine at Mount Sinai, New York, NY 10029, USA; 4 Institute of Health Informatics, University College London, London NW1 2DA, UK; 5 Health Data Research UK, Institute of Health Informatics, University College London, London NW1 2DA, UK; s.denaxas@ucl.ac.uk (S.D.); g.fatemifar@ucl.ac.uk (G.F.); 6 Institute of Cardiovascular Science, Faculty of Population Health, University College London, London WC1E 6DD, UK; c.finan@ucl.ac.uk; 7 British Heart Foundation Research Accelerator, University College London, London WC1E 6BT, UK; 8 Department of Cardiology, Division of Heart and Lungs, University Medical Center Utrecht, Heidelberglaan 100, 3584 CX Utrecht, The Netherlands; 9 UCL Genetics Institute, Division of Biosciences, University College London, London WC1E 6BT, UK

**Keywords:** CYP2C19, CYP2D6, pharmacogenetics, diabetes, personalized medicine, HbA1c, UK Biobank

## Abstract

CYP2D6 and CYP2C19 enzymes are essential in the metabolism of antidepressants and antipsychotics. Genetic variation in these genes may increase risk of adverse drug reactions. Antidepressants and antipsychotics have previously been associated with risk of diabetes. We examined whether individual genetic differences in *CYP2D6* and *CYP2C19* contribute to these effects. We identified 31,579 individuals taking antidepressants and 2699 taking antipsychotics within UK Biobank. Participants were classified as poor, intermediate, or normal metabolizers of CYP2D6, and as poor, intermediate, normal, rapid, or ultra-rapid metabolizers of CYP2C19. Risk of diabetes mellitus represented by HbA1c level was examined in relation to the metabolic phenotypes. CYP2D6 poor metabolizers taking paroxetine had higher Hb1Ac than normal metabolizers (mean difference: 2.29 mmol/mol; *p* < 0.001). Among participants with diabetes who were taking venlafaxine, CYP2D6 poor metabolizers had higher HbA1c levels compared to normal metabolizers (mean differences: 10.15 mmol/mol; *p* < 0.001. Among participants with diabetes who were taking fluoxetine, CYP2D6 intermediate metabolizers and decreased HbA1c, compared to normal metabolizers (mean difference −7.74 mmol/mol; *p* = 0.017). We did not observe any relationship between CYP2D6 or CYP2C19 metabolic status and HbA1c levels in participants taking antipsychotic medication. Our results indicate that the impact of genetic variation in CYP2D6 differs depending on diabetes status. Although our findings support existing clinical guidelines, further research is essential to inform pharmacogenetic testing for people taking antidepressants and antipsychotics.

## 1. Introduction

The use of both antidepressant and antipsychotic medications has increased steadily in recent years. Antidepressant drugs were the third most commonly prescribed drug group in 2018, with 70.9 million prescriptions across the United Kingdom—an almost two-fold increase since 2008 [1,2]. It is estimated that almost 20% of the British adult population has been prescribed an antidepressant at some stage [1,2,3]. A similar trend is seen in the prescription of antipsychotics, with an increase from eight to 12 million prescriptions between 2008 and 2018 [2]. Both antidepressant and antipsychotic medication provide essential and often lifesaving treatment for many patients. However, they are also associated with a range of common and sometimes serious adverse drug reactions including sedation, weight gain, movement disorders, and an increased risk of developing diabetes mellitus [4,5].

Most first-generation antipsychotics, as well as olanzapine and clozapine, have been shown to impair glucose regulation [5,6,7,8,9,10]. Other second generation (or atypical) antipsychotics such as amisulpride, ziprasidone, and aripiprazole seem less associated with this risk, but are still known to impair glucose regulation and increase risk of metabolic syndrome [5,6,7,8,9,10]. Several studies have linked tricyclic antidepressants to increased diabetes risk [4,11,12,13]. The evidence for selective serotonin reuptake inhibitors (SSRIs) is inconsistent, with some studies showing improved diabetic control and others showing the opposite [4,11]. Research into serotonin-noradrenaline reuptake inhibitors (SNRIs), such as venlafaxine, has reported both a lack of influence on glycemic control and diabetes risk [10,14,15,16]. Some research suggests that the risk of antidepressant-induced diabetes varies substantially between similar drugs of the same class, and thus may not be a mechanism-based adverse effect, but rather an off-target effect of a single drug [17].

Pharmacogenetics may help explain inter-individual differences in drug response and adverse drug reactions. Cytochrome P450 (CYP450) is a superfamily of enzymes involved in the oxidative biotransformation and clearance of the majority of prescribed drugs [18]. CYP2D6 and CYP2C19 are the two CYP450 enzymes most highly involved in the metabolism of antidepressant and antipsychotic drugs and are both highly polymorphic [18,19]. Genetic variation in these genes results in an altered enzyme activity and thus may explain some of the interindividual differences in treatment response. Typically, individuals are grouped into four to five phenotypic groups reflecting differing metabolic capabilities [19,20]. Poor metabolizers lack a functional enzyme due to defective or deleted genes; intermediate metabolizers usually have one functional and one defective or deleted allele causing reduced activity of the enzyme; rapid and ultra-rapid metabolizers usually have multiple copies of a functional gene or possess variants that increase gene expression [21]. Normal metabolizers (previously described as ‘extensive metabolizers’), or wild-type, are those with two fully functional copies of the gene and thus ‘normal’ enzymatic activity. The prevalence of *CYP2D6* and *CYP2C19* phenotypes varies across populations, but the extreme metabolizers are typically the least commonly observed: less than 10% of people are poor metabolizers, and less than 3% are ultra-rapid metabolizers, across all major populations and for both genes [22,23]. 

Several studies have shown that poor metabolizers of CYP2D6 or CYP2C19 have higher serum levels of antidepressants and antipsychotics, compared to normal metabolizers [24,25,26,27,28,29,30]. The Clinical Pharmacogenomics Implementation Consortium (CPIC) has developed evidence-based clinical guidelines for SSRIs and tricyclic antidepressants, recommending adjusted dosing based on *CYP2D6* and *CYP2C19* metabolic status [31,32]. There are currently no CPIC guidelines for antipsychotics, but the Dutch Pharmacogenetics Working Group provides guidelines for aripiprazole, haloperidol, pimozide and zuclopenthixol based on *CYP2D6* genotype [33]. Work to incorporate similar evidence based clinical guidelines to the UK National Health Service (NHS) is ongoing [34].

Thus far, research on the putative association between CYP450 metabolic phenotype and adverse drug reactions in response to antidepressants and antipsychotics has been limited by small sample sizes [34,35]. Little is known about pharmacogenetic influences on the diabetes risk associated with these drugs. Therefore, this study aims to examine the association between *CYP2C19* and *CYP2D6* metabolic phenotypes and the risk of diabetes mellitus in UK Biobank participants taking antidepressants and antipsychotics.

## 2. Materials and Methods

### 2.1. Sample and Phenotype Data

The UK Biobank data collection methods have been described previously in Bycroft et al. [36] and detailed study protocols are available online (http://www.ukbiobank.ac.uk/resources/, accessed on 1 September 2019 and http://biobank.ctsu.ox.ac.uk/crystal/docs.cgi/ accessed on 1 September 2019) [36,37]. The study was approved by the North-West Research Ethics Committee (ref 06/MREC08/65). All participants provided written informed consent, and those who withdrew consent after providing their sample for genetic analysis were excluded from the data extraction. Data for 502,527 UK Biobank participants were considered in this study. 

Participants were selected based on the criteria of taking one or more psychotropic drugs and were asked during a verbal interview if they were taking any ‘regular prescription medication’, and to provide the name of the medication if so. Both generic and proprietary names were recorded by UK Biobank. In these instances, we reviewed the alternative names for equivalent drugs and combined them under the generic name for analysis. For additional detail, please refer to the supplementary methods section and Supplementary Figure S1. We identified a sample of 44,051 participants taking a drug of interest. 

The UK Biobank measured a variety of biochemical markers in blood samples collected at the baseline visit. Glycated hemoglobin (HbA1c) was measured with the High Performance Liquid Chromatography (HPLC) method on a Bio-Rad VARIANT II Turbo analyzer. The HbA1c analytical range was 15–184 mmol/mol and this measurement was recorded for over 92% of the UK Biobank cohort. Data on diabetes diagnosis (self-reported and confirmed by ICD-10 diagnosis when available), antidiabetic medications, CYP2D6 and/or CYP2C19 enzyme inhibitors and body mass index (BMI) were also downloaded. Further detail is available in the supplementary methods. We identified 49 individuals who reported taking antidiabetic medication but stated that they do not have diabetes. They were excluded from the analysis due to uncertainty about their diagnosis. A total of 40,783 participants taking a psychotropic drug of interest also had HbA1c measurements available. 

### 2.2. Genetic Data and Quality Control 

The UK Biobank conducted genome-wide genotyping for 488,377 participants. Genotyping was performed using the Affymetrix UK BiLEVE Axiom array on an initial sample of 50,000 and the Affymetrix UK Biobank Axiom^®^ array (Affymetrix, Santa Clara, CA, USA) was used on all later participants [36]. These arrays include over 820,000 variants (SNPs and indel markers) and have good coverage of pharmacogenetics variants. Quality control and imputation of over 90 million variants was performed by a collaborative group led by the Wellcome Trust Centre for Human Genetics [36]. Fully imputed genetic data was downloaded in March 2018. Further local post-imputation quality control was performed in each ethnic group separately to remove variants with minor allele frequency below 1% and/or Fisher information score (a measure of the imputation accuracy for each SNP) of less than 0.3. Individuals with greater than 10% missingness, excessive genetic relatedness (greater than 10 third-degree relatives based on kinship calculations as provided centrally by UK Biobank) or mismatch between reported and genetically inferred sex were removed. 

We included both European and non-European subjects in this analysis. A list of approximately 408,000 participants of European ancestry was provided centrally by UK Biobank, based on a combination of principal component analysis (PCA) and self-reported ethnicity data [36]. Further local analysis was conducted to determine the genetic ancestry of the remaining participants: Two rounds of PCA were performed using the PC-AiR algorithm, and relatedness was estimated using PC-Relate [38,39,40,41]. This resulted in the following groups: East Asian 0.5% (*n* = 2464), South Asian 2% (*n* = 8964), African 2% (*n* = 9233) or admixed with predominantly European origin 2.5% (*n* = 11,251). A further 6686 did not cluster with any main group and were excluded from analysis. One of each pair of participants with a kinship score greater than 0.083 (approximately third-degree relatives) were excluded from the analysis. This results in a total of 40,129 participants to exclude, across all ethnicities. After these quality control procedures, a total of 33,149 participants taking antidepressant and/or antipsychotic medication with HbA1c and good quality genetic data were included in the analysis. Please see Supplementary Figure S1 for a CONSORT diagram detailing these steps. 

### 2.3. Assigning CYP Metabolic Phenotype

We extracted regions of interest for each *CYP2D6* and *CYP2C19*, defined as being one mega-base (Mb) upstream of the 5′ end of the gene and one mega-base downstream of the 3′ end of the gene (see Supplementary Table S1). Several of the SNPs of interest in this study (i.e., those that define either *CYP2D6* or *CYP2C19* star alleles) are rare (MAF < 0.01) and therefore fail standard quality control protocols. For rare SNPs of interest included on the genotype panel we used Evoker v2.4 to create intensity plots and performed visual checks to determine if the data for these SNPs was reliable enough to include [42]. We reviewed a total of six genotyped SNPs for *CYP2C19* and five for *CYP2D6*. SNPs with distinct allelic clusters were included in this study. For the rare, imputed SNPs, we included only those that met a higher Fisher information score threshold of 0.6. We reviewed a total of seven imputed SNPs for *CYP2C19* and five for *CYP2D6*. These steps enabled the inclusion of an additional four relevant SNPs for *CYP2C19*, and three for *CYP2D6*. The extraction of data and identification of rare SNPs was conducted separately for each ancestry group. 

Haplotypes for our sample were constructed based on extracted imputed genetic data using Beagle version 5.0 [43,44]. An input map and reference panel from the 1000 genome project were used [45]. The phased data was used to construct haplotypes for all participants according to the star allele nomenclature system [20,46]. We grouped individuals into *CYP2C19* metabolic phenotype groups based on the activity of the individual haplotypes and resulting diplo-types [46]. We grouped individuals into *CYP2D6* metabolic phenotype groups according to the Gaedigk activity score method [47,48]. Haplotypes containing no star-allele defining SNP variants were classified as wild-type (*1, please see [20] and [46] for more detail on the star-allele nomenclature system) alleles for the corresponding gene. Because not all star allele-defining SNPs were available in our genetic dataset, we expect a fraction of haplotypes to be misclassified as wild-type. Nonetheless, as the cumulative reported frequency of the missing SNPs is very low, we expect the number of misclassified haplotypes to be small. In addition, we did not have data on *CYP2D6* copy number variants (CNVs). This means we are not able to define *CYP2D6* ultra-rapid metabolizers, or other whole gene deletions (e.g., CYP2D6*5).

### 2.4. Statistical Analysis

We conducted a grouped analysis of all tricyclic antidepressants, as previous evidence suggests that they all cause an increase in HbA1c to some extent [49]. We did not analyze SSRIs as a group due to variable evidence on their influence on HbA1c in the literature [15,17,49]. Any antidepressants taken by over 1800 participants were analyzed independently (amitriptyline, citalopram, fluoxetine, sertraline, paroxetine, venlafaxine). Medications were grouped according to whether their primary metabolic pathway was catalyzed by CYP2D6 or CYP2C19, based on the Maudsley Prescribing Guidelines and CPIC guidelines [10,31,32]. Tricyclic antidepressants that are known CYP2C19 substrates are: amitriptyline, clomipramine, doxepin, imipramine and trimipramine. SSRIs that are known CYP2C19 substrates are citalopram, escitalopram, and sertraline. Tricyclic antidepressants that are known substrates for CYP2D6 include amitriptyline, clomipramine, duloxetine, and doxepin. SSRIs that are known substrates for CYP2D6 are fluoxetine, fluvoxamine, paroxetine, sertraline, as well as the SNRIs mirtazapine and venlafaxine [10,50]. Several drugs are metabolized through both CYP2C19 and CYP2D6 (e.g., tricyclic antidepressants). In these cases, the metabolic phenotypes of both genes were included in the same analyses.

No single antipsychotic drug had sufficient sample size to allow for individual analysis. Therefore, we included all antipsychotic drugs known to be metabolized at least in part by CYP2D6: aripiprazole, clozapine, fluphenazine, haloperidol, olanzapine, perphenazine, pimozide, risperidone, zuclopenthixol, thioridazine. CYP2C19 does not play a significant role in the metabolism of antipsychotics [10].

For each drug or drug group, we ran linear regression models with HbA1c as the outcome of interest and CYP450 metabolic phenotype and diabetes status as the main explanatory variables. All statistical models were adjusted to account for any participant taking antidiabetic treatment or taking drugs, psychotropic or otherwise, that are known inhibitors of the enzymes of interest. Additional covariates included were BMI, sex, age, and genetically determined ancestry group. We investigated the interaction of diabetes status and CYP metabolic phenotype. Where this interaction was significant (*p* < 0.05) we conducted a stratified analysis separating participants into two groups based on their diabetes status.

Some of these analyses are nested (individual drug analyses overlap with drug group analyses), and, as such, we concluded that a Bonferroni correction for multiple testing would be excessively stringent [51]. Therefore, we report uncorrected *p* values in all text and tables, but as recommended by Li et al. (2012) [52], we have an adjusted significance threshold of *p* < 0.05/2 = 0.025 (threshold for a suggestive association *p* < 0.1/2 = 0.05) for the two grouped analyses, and *p* < 0.05/6 = 0.0083 (threshold for a suggestive association *p* < 0.1/6 = 0.017) for the individual drug analyses examining six specific drugs. All statistical analyses were performed using R version 3.6.0 [53,54,55]. 

## 3. Results

### 3.1. Dataset

We identified 33,149 UK Biobank participants who reported taking at least one antidepressant or antipsychotic and had HbA1c and genetic data passing quality control (antidepressants *n* = 31,579, antipsychotics *n* = 2699) (Table 1). Our sample included 22,632 (68.3%) females and 10,517 (31.7%) males (see Table 1). Mean age was 56.6 ± 7.8 years, range 40 to 70 years. Full demographic data and summary statistics of our sample are shown in the Table 1 (see also Supplementary Tables S5 and S6). 

### 3.2. Psychotropics Prescribed in the UK Biobank

**Figure 1:**
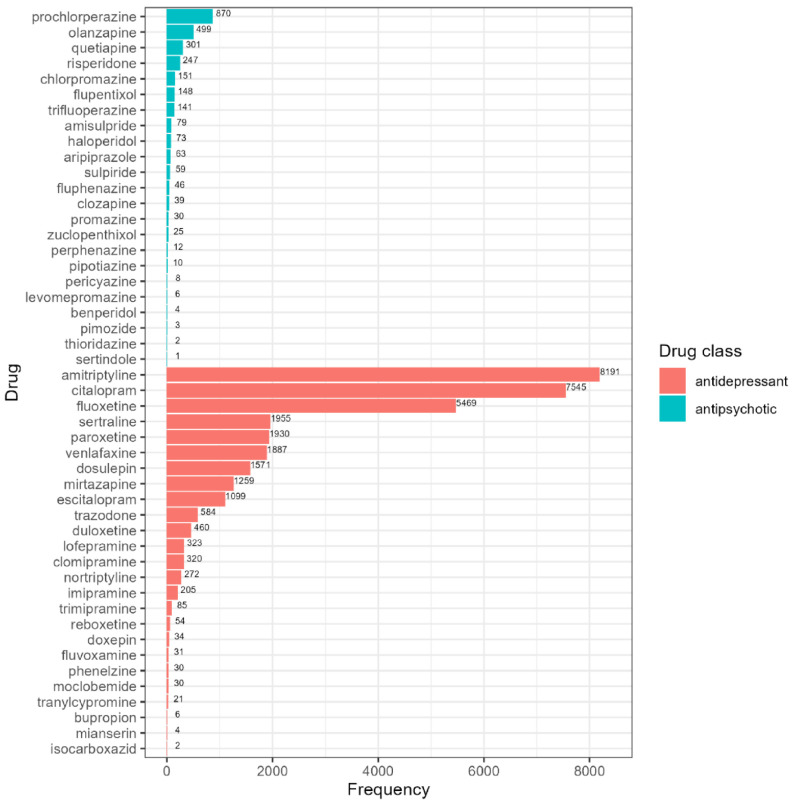
Frequency table of identified antipsychotics (blue bars) and antidepressants (red bars) in UK Biobank.

There were 28 different antidepressants identified in our sample (Figure 1; Supplementary Tables S7 and S8). Amitriptyline was the most common drug in our cohort (*n* = 8191). We identified 24 different antipsychotic drugs (Figure 1; Supplementary Table S9), with the most frequent antipsychotics being prochlorperazine (870 individuals, 30.9%), followed by olanzapine (499 individuals, 17.7%). Among UK Biobank participants taking antidepressants, 5.2% report taking more than one different antidepressant concurrently (of these, 2% report taking three or four). Of those taking antipsychotics, 4.5% report taking more than one different antipsychotic medication concurrently (of these, 7.4% report taking three or four). The co-prescription of an antidepressant with antipsychotics is very common, with 41.4% of subjects taking antipsychotics also taking at least one antidepressant. 

The included covariates (diabetes status, antidiabetic medications, BMI, age, sex, and ethnicity) affected HbA1c as expected. Please refer to the supplementary methods for further details.

### 3.3. Antidepressants and CYP Metabolic Status

**Figure 2:**
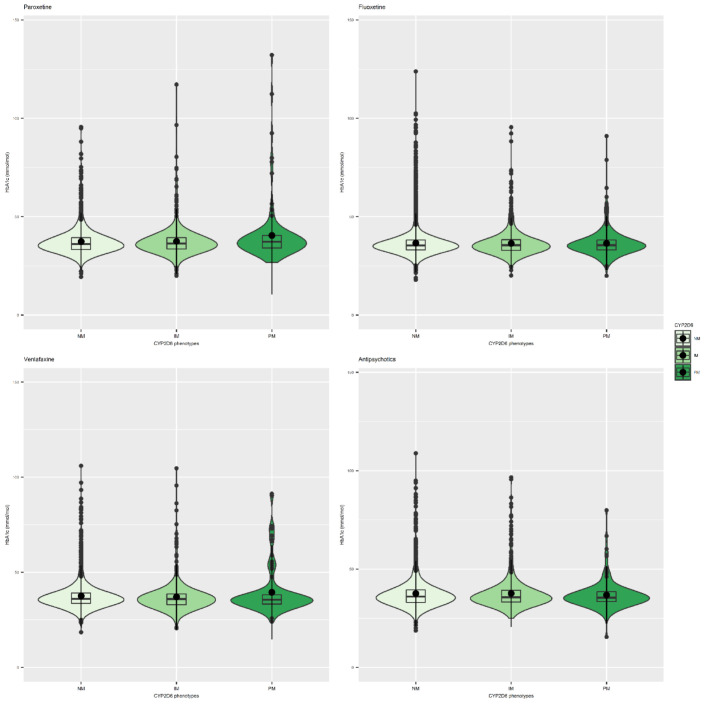
Violin plots showing the relationship between CYP2D6 metabolic status and HbA1c levels (mmol/mol) among subjects taking (from left to right) paroxetine, fluoxetine, venlafaxine, and all antipsychotics.

For several of the antidepressants investigated, we consistently found that the interaction of diabetes status and *CYP2D6* and *CYP2C19* metabolic phenotype is statistically significant (Supplementary Figure S2). Where this was the case, we stratified our analyses by whether participants had diabetes or not. We observed this interaction for fluoxetine, venlafaxine, citalopram, sertraline, and amitriptyline, and for tricyclic antidepressants as a class. Among all participants (regardless of diabetes status) taking paroxetine (SSRI), we observe significantly higher HbA1c levels among *CPY2D6* poor metabolizers (mean difference: 2.43 mmol/mol; 95% CI (1.23,3.63); *p* = 7.77 × 10^−5^) (see Table 2, Figure 2, and Supplementary Table S10). A stratified analysis of diabetic participants taking fluoxetine (SSRI) reveals a suggestive association between *CYP2D6* intermediate metabolizers and lower HbA1c levels compared to normal metabolizers (mean difference = −3.74 mmol/mol; 95% CI [−6.82, −0.67]; *p* = 0.017 (see Table 3 and Table 4, Figure 2, and Supplementary Table S11). In participants taking venlafaxine (SNRI), we found that, amongst people with diabetes, poor metabolizers for *CYP2D6* had higher HbA1c than normal metabolizers (mean difference: 10.15mmol/mol; 95% CI (2.63,17.67); *p* = 0.008) (see Table 5 and Table 6, Figure 2, and Supplementary Table S12).

Stratified analyses of citalopram and sertraline did not reveal any significant association between *CYP2C19* metabolic status and HbA1c levels (see Supplementary Tables S13–S16). Stratified analysis of amitriptyline did not reveal any significant association between either *CYP2C19* or *CYP2D6* metabolic status and HbA1c levels (see Supplementary Tables S17 and S18).

Several tricyclic antidepressants were reported too infrequently to allow for single-drug analysis. Therefore, we grouped the remaining drugs of this class, excluding amitriptyline as its higher frequency would have heavily driven the findings. We again stratified the group based on diabetes status and found no significant associations between either *CYP2C19* or *CYP2D6* derived metabolic groups and HbA1c (see Supplementary Tables S19 and S20).

We did not observe any significant association between HbA1c levels and *CYP2C19* metabolic status in individuals taking antidepressants. In addition, we find that participants taking drugs that act as *CYP2C19* inhibitors, regardless of *CYP2C19* metabolic status, experience higher levels of HbA1c. Citalopram: mean difference: 0.36 mmol/mol, 95% CI (0.07,0.65); *p* = 0.016); Amitriptyline: mean difference: 0.37 mmol/mol; 95% CI (0.09,0.64); *p* = 0.009; Tricyclics: mean difference = 0.39 mmol/mol; 95% CI (0.13,0.66); *p* = 0.004). We did not see this relationship with sertraline (see Supplementary Tables S13, S15, S17 and S19).

### 3.4. Antipsychotics and CYP Metabolic Status

We find no evidence that the metabolic phenotypes of CYP2D6 influence HbA1c levels amongst 2699 people taking antipsychotic medications. Similarly, taking a CYP2D6 inhibitor drug was not significantly associated with HbA1c levels amongst people taking antipsychotic medication. This was the case in the full sample of all people taking antipsychotics, of whom 40% also take an antidepressant, and in a sub-analysis including participants who only take antipsychotics. See Table 7, Figure 2 and Supplementary Tables S21 and S22.

## 4. Discussion

Non-normal metabolic phenotypes of CYP2D6 and CYP2C19 have been linked to QT prolongation [57,58], weight gain [56,59,60,61], hormonal changes among patients taking psychotropic medication, and increased risk of extrapyramidal adverse reactions to antipsychotics [62]. However, recent studies and meta-analyses have yielded inconclusive or negative findings and the clinical significance of CYP450 metabolic phenotypes is still in question [30,63]. Several studies agree that long-term antidepressant treatment increases risk of developing diabetes [4,64,65,66], but the extent to which this specific adverse drug reaction is impacted by genetics is unknown. To our knowledge, this study is the first to explore if variation in the *CYP2D6* and *CYP2C19* genes influences HbA1c levels in individuals taking antidepressants and antipsychotics. Most previous studies of CYP450 metabolic status and adverse drug reactions are limited by small sample sizes and low representation of the less common poor or ultra-rapid metabolizers [30,56,57,58,59,60,61,62,63,64,65,66]. This study represents one of the largest available samples of individuals taking antidepressants and antipsychotics and includes a much higher number of extreme CYP450 metabolizers than seen in previous publications (*n* = 9878 non-wild-type *CYP2D6* metabolizers and *n* = 21,273 non-wild-type *CYP2C19* metabolizers). The reported frequencies of the included drugs in this sample (Figure 1) are broadly consistent with UK prescribing patterns of psychotropic medication, although it is worth noting that amitriptyline (the most common antidepressant in our sample) is more frequently prescribed to treat anxiety and sleep problems [67].

We find a significant association between *CYP2D6* poor metabolizers and higher levels of HbA1c among all participants taking paroxetine with an average increase of 2.3 mmol/mol, a substantial effect. The Clinical Pharmacogenetics Implementation Consortium (CPIC) guidelines recommend using lower doses of paroxetine for poor metabolizers of *CYP2D6* [32]. Thus, our findings are consistent with existing pharmacokinetic evidence and provide further support for the CPIC guidelines. Of interest, some research found that prolonged use of paroxetine was associated with phenocopying, an environmentally induced conversion of normal metabolizers to poor metabolizers [68,69,70].

We observe a significant interaction between diabetic status and non-wild-type CYP status for participants taking amitriptyline, fluoxetine, citalopram, sertraline, and venlafaxine. We conducted stratified analyses of these drugs and found suggestive evidence that, in diabetic participants taking venlafaxine, *CYP2D6* poor and intermediate metabolizers have higher HbA1c levels. Like paroxetine, venlafaxine has been previously associated with an increased risk of diabetes [4,15,71]. Our study finds that diabetic *CYP2D6* poor metabolizers treated with venlafaxine have on average 10.15 mmol/mol higher HbA1c levels than diabetic normal metabolizers. Though this is a suggestive association only with a comparatively small sample size, it is consistent with the guidelines published by the Dutch Pharmacogenetics Working Group which suggest that *CYP2D6* poor metabolizers should be treated with an alternative antidepressant or have their venlafaxine dose reduced [33]. In addition, a stratified analysis reveals suggestive evidence that diabetic *CYP2D6* intermediate metabolizers taking fluoxetine have lower HbA1c levels compared to diabetic *CYP2D6* normal metabolizers. Although this is contrary to our initial hypothesis, there is some evidence to suggest that fluoxetine can lower HbA1c levels in diabetic patients, despite increasing risk of type 2 diabetes in non-diabetic patients [72,73,74]. Our findings add support to this theory, suggesting that decreased *CYP2D6* metabolism may in fact be somewhat beneficial for patients with diabetes who take fluoxetine.

Contrary to our hypotheses, we did not find evidence of associations between *CYP2D6* or *CYP2C19* metabolic status and HbA1c in people treated with amitriptyline and other tricyclics. Although CPIC guidelines exist for *CYP2C19* and *CYP2D6* poor metabolizers taking tricyclic antidepressants, they state that suggested dose alterations or treatment changes are optional based on the limited strength of existing evidence [31]. Our analyses of tricyclics antidepressants and amitriptyline alone were adequately powered with over 400 poor metabolizers of each gene, making it one of the largest samples of abnormal CYP metabolizers available. However, the metabolic pathway of amitriptyline (and other tertiary amine tricyclic antidepressants) involves two steps: the first step is catalyzed by CYP2C19 and produces an active metabolite (nortriptyline). The second step is the metabolism of nortriptyline to an inactive metabolite, via CYP2D6 [75,76]. For this reason, we included the metabolic phenotypes of both CYP2C19 and CYP2D6 in the analysis. Despite this, it is likely that pairing these analyses with dose data, or ideally serum drug level data, would be necessary to fully elucidate the extent of the synergistic action of CYP2D6 and CYP2C19 on amitriptyline metabolism.

In addition, we did not find associations between *CYP2D6* variation and HbA1c amongst people taking antipsychotics, nor did we observe an impact of CYP2D6 inhibitors. Given the total sample size of 2699, we undertook a combined analysis including all antipsychotics, which have various levels of influence on glucose regulation and diabetes risk. Although this sample is the largest available with 135 *CYP2D6* poor metabolizers overall, statistical power remains limited given the heterogeneity of the sample. There is a great deal of variation between the included drugs in this study, both in terms of their mechanism of action and in their relative risk of glucose dysregulation. Future study in a larger sample would allow for the separate analysis of all individual drugs and should yield more conclusive results. This limitation also applies to the less common antidepressants in our sample, which were included in grouped analyses only. Given that UK Biobank is a population study, utilizing existing data from large patient-based biobanks such as the Million Veteran Program could be a valuable continuation of this work [77]. Biobanks from countries with more historically isolated populations, such as Finngen, may contain a higher proportion of some rare SNPs that are necessary to define additional CYP450 star alleles.

As well as being impacted by genetic variation, CYP2D6 and CYP2C19 enzyme activity is susceptible to inhibition by other compounds. We observed that taking CYP2C19 inhibitors (of which proton pump inhibitors were the most common in our sample) led to higher HbA1c levels in people taking tricyclic antidepressants, amitriptyline, and citalopram. Thus, based on our data, there is substantial potential for drug interactions and drug–drug–CYP2C19 interactions. These should be investigated further and considered for inclusion in future clinical guidelines. We did not find evidence that taking CYP2D6 inhibitors affected HbA1c levels in people taking antipsychotics or antidepressants. This enzyme inhibition could, however, still be important for other psychotropic adverse effects such as QT prolongation. It is also worth noting that CYP2C19 inhibitor drugs were taken by approximately a quarter of the subjects included in these analyses. That may have decreased the power to detect a significant association between the (genetically determined) *CYP2C19* metabolic status and HbA1c levels, because if the inhibitory impact of a drug was strong enough it would reduce or eliminate the impact of the genetic variation, i.e., an *CYP2C19* normal metabolizer taking an inhibitor may have the same enzymatic activity and as *CYP2C19* poor metabolizer taking an inhibitor. Future analyses with larger sample sizes should investigate this interaction further.

A clear limitation of this study is the reliance on certain self-reported data (including diabetes diagnosis). In addition, we have used only the baseline, cross-sectional UK Biobank data and therefore lack detail on treatment dose and duration. Most adverse drug reactions to antidepressants and antipsychotics are dose-dependent, and thus further analysis including this data is warranted. Besides, diabetes is a complex disease with many genetic and environmental risk factors. We do account for several confounding environmental factors in our analyses, including whether subjects are taking antidiabetic medication. However, it is possible that additional factors such as the co-prescription of non-antidiabetic agents that can alter glucose regulation could be impacting the results [78]. Although the SNP-based heritability of diabetes is estimated to be less than 20%, the inclusion of polygenic risk scores for diabetes may improve analyses of pharmacogenetic associations by capturing background genetic disease risk [79]. A genome-wide gene–environment interaction study may also highlight other genes of potential interest. Finally, although we included participants of all ethnicities in this analysis, UK Biobank is predominantly European. There is a great deal of variation in the frequency of functional variants within the CYP450 genes across different populations [22,80], as well as in the risk of diabetes. The field of pharmacogenetics would be greatly benefitted by further study in more diverse samples.

Although both arrays used by UK Biobank have relatively good coverage of *CYP2C19* and *CYP2D6*, several SNPs that define known star alleles were neither genotyped nor imputed, nor otherwise met the criteria for inclusion as described in the methods. Therefore, we expect a small number of individuals to be misclassified as normal metabolizers. However, we anticipate this number to be small given the low minor allele frequency of the missing variants. We were unable to include *CYP2D6* ultra-rapid metabolizers in this study, as copy number and other structural variants were not defined. *CYP2D6* ultra-rapid metabolizers are the least common phenotypic group across all populations, with a frequency of less than 2% in European, South Asian, East Asian and Admixed European groups, and approximately 3–6% in African ancestry groups [22,80]. *CYP2D6* ultra-rapid metabolizers therefore represent a very small minority in our sample, and they have been combined with the normal metabolizers group by default. We estimate this to have a small effect on our results as we would expect ultra-rapid metabolizers to be less susceptible to adverse drug reactions, though it will be important to consider this group in future studies of treatment failure. The availability of whole genome sequencing data will improve the accuracy with which highly polymorphic pharmaco-genes like *CYP2D6* can be characterised, whilst still capturing the important splicing or non-coding variants that may be missed with exome sequencing data [81].

## 5. Conclusions

Overall, our findings are broadly consistent with existing guidelines for antidepressants and point towards the necessity of including more antidepressants and antipsychotics in pharmacogenetic clinical trials and experimental medicine studies. These results also suggest that there is a need for randomized double-blinded clinical trials to further explore genetic testing as a guide to antidepressant/antipsychotic treatment. Indeed, studies show that pharmacogenetic testing is practical [82], accurately predicts the outcomes of antidepressant treatments [83] and improves outcomes [84,85]. It has also been demonstrated that it can reduce the total cost of antipsychotic treatment by 28% [86]. Findings from this study need to be followed up with further longitudinal testing, with a focus on singular antidepressants and antipsychotics, more adverse drug reactions, and in more diverse populations.

## Figures and Tables

**Table 1:** Demographic Data for Study Sample.

	Antidepressants (*n* = 31,579)	Antipsychotics (*n* = 2699)
*CYP2D6* metabolic phenotype		
Normal metabolizers	22,486 (71.2%)	1914 (70.9%)
Intermediate metabolizers	7433 (23.5%)	650 (24.1%)
Poor metabolizers	1660 (5.3%)	135 (5.0%)
*CYP2C19* metabolic phenotype		
Normal metabolizers	12,001 (38.0%)	1004 (37.2%)
Intermediate metabolizers	9367 (29.7%)	789 (29.2%)
Poor metabolizers	1065 (3.4%)	100 (3.7%)
Rapid metabolizers	7805 (24.7%)	686 (25.4%)
Ultra-rapid metabolizers	1341 (4.2%)	120 (4.4%)
Takes CYP2D6 inhibitors ^A^		
No	29,713 (94.1%)	2548 (94.4%)
Yes	1866 (5.9%)	151 (5.6%)
Takes CYP2C19 inhibitors ^A^		
No	23,608 (74.8%)	2091 (77.5%)
Yes	7971 (25.2%)	608 (22.5%)
Sex		
Female	21,752 (68.9%)	1553 (57.5%)
Male	9827 (31.1%)	1146 (42.5%)
Age		
Mean (SD) (years)	56.6 (7.78)	56.4 (8.12)
Range (median) (years)	40–70 (58)	40–70 (57)
Ethnicity		
Caucasian	29,628 (93.8%)	2403 (89.0%)
Admix Caucasian	795 (2.5%)	72 (2.7%)
African	289 (0.9%)	90 (3.3%)
East Asian	43 (0.1%)	12 (0.4%)
Other	450 (1.4%)	57 (2.1%)
South Asian	374 (1.2%)	65 (2.4%)
Hb1Ac		
Mean (SD) (mmol/mol)	37.1 (7.75)	37.5 (8.31)
Diabetes status		
No	28,776 (91.1%)	2415 (89.5%)
Yes	2803 (8.9%)	284 (10.5%)
Takes antidiabetic medications ^B^		
No	29,573 (93.6%)	2491 (92.3%)
Yes	2006 (6.4%)	208 (7.7%)
BMI		
Mean (SD) (kg/m^2^)	28.8 (5.66)	29.1 (5.94)

^A^ CYP2C19 and CYP2D6 inhibitors identified through review of literature, including British National Formulary; ^B^ As defined by British National Formulary [56].

**Table 2:** Association between CYP2D6 metabolic phenotype and HbA1c levels among participants taking paroxetine. Model adjusted by age, ethnicity, sex, taking inhibitors of CYP2D6, diabetes status, taking antidiabetics and BMI; Normal metabolizers of CYP2D6 taking paroxetine: 1367.

Predictors	Paroxetine			
	*n*	Estimates	CI	*p*
Diabetes	174	6.85	5.11, 8.59	<0.001
CYP2D6 IM	457	0.23	−0.42, 0.87	0.489
CYP2D6 PM	106	2.43	1.23, 3.63	<0.001
Observations	1930			
R^2^/R^2^ adjusted	0.454/0.450			

**Table 3:** Association between CYP2D6 metabolic phenotype and HbA1c levels among participants taking fluoxetine. Model adjusted by age, ethnicity, sex, taking inhibitors of CYP2D6, diabetes status, taking antidiabetics and BMI; Normal metabolizers of CYP2D6: 3888.

			Fluoxetine		
Predictors	*n*	Estimates		CI	*p*
Diabetes	426	7.22		6.20, 8.23	<0.001
CYP2D6 IM	1282	0.06		−0.29, 0.41	0.728
CYP2D6 PM	299	0.04		−0.62, 0.69	0.916
Diabetes: CYP2D6 IM		−3.78		−5.03, −2.53	<0.001
Diabetes: CYP2D6 PM		−1.81		−4.11, 0.49	0.124
Observations			5469		
R^2^/R^2^ adjusted			0.467/0.465		

**Table 4:** Stratified analysis of diabetes status among participants taking fluoxetine. Model adjusted by age, ethnicity, sex, Table 2. D6, taking antidiabetics and BMI; Normal metabolizers of CYP2D6: diabetes = 302.

			Diabetes			No Diabetes		
Predictors	*n*	Estimates	CI	*p*	*n*	Estimates	CI	*p*
CYP2D6 IM	100	−3.74	−6.82, −0.67	0.017	1182	0.05	−0.21, 0.31	0.696
CYP2D6 PM	24	−0.94	−6.61, 4.73	0.745	275	0.04	−0.43, 0.52	0.859
Observations			426			5043		
R^2^/R^2^ adjusted			0.196/0.175			0.130/0.128		

**Table 5:** Association between CYP2D6 metabolic phenotype and HbA1c levels among participants taking venlafaxine. Model adjusted by age, ethnicity, sex, taking inhibitors of CYP2D6, diabetes status, taking anti-diabetics and BMI; Normal metabolizers of CYP2D6: 1352.

			Venlafaxine		
Predictors	*n*	Estimates		CI	*p*
Diabetes	182	5.68		4.04, 7.33	1.77 × 10^−11^
CYP2D6 IM	430	−0.23		−0.89, 0.43	0.495
CYP2D6 PM	103	−0.46		−1.73, 0.80	0.473
Diabetes: CYP2D6 IM		3.62		1.27, 5.98	0.003
Diabetes: CYP2D6 PM		11.44		8.05, 14.84	4.79 × 10^−11^
Observations			1887		
R^2^/R^2^ adjusted			0.528/0.524		

**Table 6:** Stratified analysis of diabetes status among participants taking venlafaxine. Model adjusted by age, ethnicity, sex, taking inhibitors of CYP2D6, taking antidiabetics and BMI; Normal metabolizers of CYP2D6: diabetes = 135.

			Diabetes			No Diabetes		
Predictors	*n*	Estimates	CI	*p*	*n*	Estimates	CI	*p*
CYP2D6 IM	32	3.55	−1.75, 8.85	0.188	398	−0.22	−0.71, 0.26	0.367
CYP2D6 PM	15	10.15	2.63, 17.67	0.008	88	−0.44	−1.36, 0.49	0.356
Observations			182			1703		
R^2^/R^2^ adjusted			0.280/0.233			0.122/0.116		

**Table 7:** Association between CYP2D6 metabolic phenotype and HbA1c levels in participants taking antipsychotics. Model adjusted by age, ethnicity, sex, taking inhibitors of CYP2D6, diabetes status, taking antidiabetics and BMI; Normal metabolizers of CYP2D6 = 1914.

Predictors	*n*/mean	HbA1c mmol/mol Estimates	95% CI mmol/mol	*p*
CYP2D6 IM	650	−0.02	−0.58, 0.53	0.930
CYP2D6 PM	135	−0.93	−2.01, 0.16	0.093
Takes CYP2D6 inhibitor	151	0.59	−0.43, 1.61	0.260
Diabetes	284	4.55	3.13, 5.97	<0.001
Observations	2699			
R^2^ / R^2^ adjusted	0.449/0.446			

## Data Availability

All data used in this study is publicly available to authorized researched via the UK Biobank: https://www.ukbiobank.ac.uk/, accessed on 1 September 2021. Detail on the available data can be found here: https://biobank.ndph.ox.ac.uk/showcase/, accessed on 1 September 2021.

## Supplementary Materials

The following are available online at https://www.mdpi.com/article/10.3390/genes12111758/s1. Supplementary methodology. Figure S1: Adapted CONSORT 2010 statement. Figure S2: Interaction between diabetes status and metabolic phenotypes among subjects taking, from left to right, (a) tricyclic antidepressants; (b) Amitriptyline; (c) Fluoxetine; (d) Venlafaxine; (e) Citalopram; (f) Sertraline. Table S1: Showing start and stop positions used to extract relevant genetic data from full UKB sample. Table S2: Frequencies of *CYP2C19* diplo-types and metabolic phenotypes in 33,149 Biobank participants taking antidepressants or antipsychotics. Table S3: Frequencies of *CYP2D6* diplo-types and metabolic phenotypes in 33,149 Biobank participants taking antidepressants or antipsychotics. Table S4: HbA1c levels and CYP phenotypes across individual and groups of medications. Table S5: Characteristics of CYP2C19 metabolic phenotype in our sample. Table S6: Characteristics of CYP2D6 metabolic phenotype in our sample. Table S7: CYP2D6 metabolic phenotypes of people taking antidepressants. Table S8: CYP2C19 metabolic phenotypes of people taking antidepressants. Table S9: CYP2D6 metabolic phenotype of individuals taking antipsychotics. Table S10: Association between CYP2D6 metabolic phenotype and HbA1c within individuals taking paroxetine—Additional detail. Table S11: Association between CYP2D6 metabolic phenotype and HbA1c within individuals taking fluoxetine—additional detail. Table S12: Association between CYP2D6 metabolic phenotype and HbA1c within individuals taking venlafaxine—additional detail. Table S13: Association between CYP2C19 metabolic phenotype and HbA1c within individuals taking citalopram. Table S14: Stratified analysis of people taking citalopram. Table S15: Association between CYP2C19 metabolic phenotype and HbA1c within individuals taking sertraline. Table S16: Stratified analysis of people taking sertraline. Table S17: Association between CYP2D6 and CYP2C19 metabolic phenotype and HbA1c within Amitriptyline. Table S18: Stratified analysis of people taking amitriptyline. Table S19: Association between CYP2D6 and CYP2C19 metabolic phenotype and HbA1c within individuals taking tricyclic antidepressants. Table S20: Stratified analysis of people taking tricyclic antidepressants. Table S21: Antipsychotics regression model. Association between CYP2D6 metabolic phenotype and HbA1c—additional detail. Table S22: Association between CYP2D6 metabolic phenotype and HbA1c among participants taking only antipsychotics, without a co-prescribed antidepressant.

## References

[1] Taylor S., Annand F., Burkinshaw P., Greaves F., Kelleher M., Knight J., Perkins C., Tran A., White M., Marsden J. (2019). Dependence and Withdrawal Associated with Some Prescribed Medicines: An Evidence Review.

[2] (2018). Prescription Cost Analysis—England. https://digital.nhs.uk/data-and-information/publications/statistical/prescription-cost-analysis/2018.

[3] Iacobucci G. (2019). NHS prescribed record number of antidepressants last year. BMJ.

[4] Andersohn F., Schade R., Suissa S., Garbe E. (2009). Long-Term Use of Antidepressants for Depressive Disorders and the Risk of Diabetes Mellitus. Am. J. Psychiatry.

[5] Holt R.I.G. (2019). Association Between Antipsychotic Medication Use and Diabetes. Curr. Diabetes Rep..

[6] American Diabetes Association, American Psychiatric Association, American Association of Clinical Endocrinologists, North American Association for the Study of Obesity (2004). Consensus Development Conference on Antipsychotic Drugs and Obesity and Diabetes. Diabetes Care.

[7] Gill S.P. (2005). Stable monotherapy with clozapine or olanzapine increases the incidence of diabetes mellitus in people with schizophrenia. Evid. Based Ment. Health.

[8] Leslie D.L., Rosenheck R.A. (2004). Incidence of newly diagnosed diabetes attributable to atypical antipsychotic medications. Am. J. Psychiatry.

[9] Schwenkreis P., Assion H.-J. (2004). Atypical antipsychotics and diabetes mellitus. World J. Biol. Psychiatry.

[10] Taylor D., Barnes T., Young A. (2018). The Maudsley Prescribing Guidelines in Psychiatry.

[11] Kivimäki M., Hamer M., Batty G.D., Geddes J.R., Tabak A.G., Pentti J., Virtanen M., Vahtera J. (2010). Antidepressant medication use, weight gain, and risk of type 2 diabetes: A population-based study. Diabetes Care.

[12] Lustman P.J., Griffith L.S., Clouse R.E., Freedland K.E., Eisen S.A., Rubin E.H., Carney R.M., McGill J.B. (1997). Effects of Nortriptyline on Depression and Glycemic Control in Diabetes: Results of a Double-blind, Placebo-controlled Trial. Psychosom. Med..

[13] Mumoli N., Cocciolo M., Vitale J., Mantellassi M., Sabatini S., Gambaccini L., Cei M. (2014). Diabetes mellitus associated with clomipramine treatment: A retrospective analysis. Acta Diabetol..

[14] Burcu M., Zito J.M., Safer D.J., Magder L.S., dos Reis S., Shaya F.T., Rosenthal G.L. (2017). Association of Antidepressant Medications with Incident Type 2 Diabetes Among Medicaid-Insured Youths. JAMA Pediatr..

[15] Gagnon J., Lussier M.-T., MacGibbon B., Daskalopoulou S.S., Bartlett G. (2018). The Impact of Antidepressant Therapy on Glycemic Control in Canadian Primary Care Patients with Diabetes Mellitus. Front. Nutr..

[16] Hall J.A., Wang F., Oakes T.M.M., Utterback B.G., Crucitti A., Acharya N. (2010). Safety and tolerability of duloxetine in the acute management of diabetic peripheral neuropathic pain: Analysis of pooled data from three placebo-controlled clinical trials. Expert Opin. Drug Saf..

[17] Barnard K., Peveler R.C., Holt R.I.G. (2013). Antidepressant medication as a risk factor for type 2 diabetes and impaired glucose regulation: Systematic review. Diabetes Care.

[18] Zanger U.M., Schwab M. (2013). Cytochrome P450 enzymes in drug metabolism: Regulation of gene expression, enzyme activities, and impact of genetic variation. Pharmacol. Ther..

[19] Ingelman-Sundberg M. (2005). Genetic polymorphisms of cytochrome P450 2D6 (CYP2D6): Clinical consequences, evolutionary aspects and functional diversity. Pharm. J..

[20] Sim S.C., Ingelman-Sundberg M. (2010). The Human Cytochrome P450 (CYP) Allele Nomenclature website: A peer-reviewed database of CYP variants and their associated effects. Hum. Genom..

[21] Ingelman-Sundberg M., Sim S.C., Gomez A., Rodriguez-Antona C. (2007). Influence of cytochrome P450 polymorphisms on drug therapies: Pharmacogenetic, pharmacoepigenetic and clinical aspects. Pharmacol. Ther..

[22] Gaedigk A., Sangkuhl K., Whirl-Carrillo M., Klein T., Leeder J.S. (2017). Prediction of CYP2D6 phenotype from genotype across world populations. Genet. Med. Off. J. Am. Coll. Med. Genet..

[23] Martis S., Peter I., Hulot J.-S., Kornreich R., Desnick R.J., Scott S.A. (2013). Multi-ethnic distribution of clinically relevant CYP2C genotypes and haplotypes. Pharm. J..

[24] Grasmäder K., Verwohlt P.L., Rietschel M., Dragicevic A., Müller M., Hiemke C., Freymann F.N., Zobel A., Maier W., Rao M.L. (2004). Impact of polymorphisms of cytochrome-P450 isoenzymes 2C9, 2C19 and 2D6 on plasma concentrations and clinical effects of antidepressants in a naturalistic clinical setting. Eur. J. Clin. Pharmacol..

[25] Chang M., Tybring G., Dahl M.-L., Lindh J.D. (2014). Impact of cytochrome P450 2C19 polymorphisms on citalopram/escitalopram exposure: A systematic review and meta-analysis. Clin. Pharmacokinet..

[26] Huezo-Diaz P., Perroud N., Spencer E.P., Smith R., Sim S., Virding S., Uher R., Gunasinghe C., Gray C., Campbell D. (2012). CYP2C19 genotype predicts steady state escitalopram concentration in GENDEP. J. Psychopharmacol..

[27] Chen R., Wang H., Shi J., Shen K., Hu P. (2015). Cytochrome P450 2D6 genotype affects the pharmacokinetics of controlled-release paroxetine in healthy Chinese subjects: Comparison of traditional phenotype and activity score systems. Eur. J. Clin. Pharmacol..

[28] Rudberg I., Hermann M., Refsum H., Molden E. (2008). Serum concentrations of sertraline and N-desmethyl sertraline in relation to CYP2C19 genotype in psychiatric patients. Eur. J. Clin. Pharmacol..

[29] Schenk P.W., van Vliet M., Mathot R.A., van Gelder T., Vulto A.G., van Fessem M.A.C., Rij S.V.-V., Lindemans J., Bruijn J.A., van Schaik R.H.N. (2010). The CYP2C19*17 genotype is associated with lower imipramine plasma concentrations in a large group of depressed patients. Pharm. J..

[30] Milosavljevic F., Bukvic N., Pavlovic Z., Miljevic C., Pešic V., Molden E., Ingelman-Sundberg M., Leucht S., Jukić M.M. (2021). Association of CYP2C19 and CYP2D6 Poor and Intermediate Metabolizer Status with Antidepressant and Antipsychotic Exposure: A Systematic Review and Meta-analysis. JAMA Psychiatry.

[31] Hicks J.K., Sangkuhl K., Swen J.J., Ellingrod V.L., Müller D.J., Shimoda K., Bishop J.R., Kharasch E.D., Skaar T.C., Gaedigk A. (2016). Clinical pharmacogenetics implementation consortium guideline (CPIC) for CYP2D6 and CYP2C19 genotypes and dosing of tricyclic antidepressants: 2016 update. Clin. Pharm. Ther..

[32] Hicks J.K., Bishop J.R., Sangkuhl K., Muller D.J., Ji Y., Leckband S.G., Leeder J.S., Graham R.L., Chiulli D.L., LLerena A. (2015). Clinical Pharmacogenetics Implementation Consortium (CPIC) guideline for CYP2D6 and CYP2C19 genotypes and dosing of selective serotonin reuptake inhibitors. Clin. Pharmacol. Ther..

[33] Swen J.J., Nijenhuis M., de Boer A., Grandia L., Maitland-van der Zee A.H., Mulder H., Rongen G.A., van Schaik R.H., Schalekamp T., Touw D.J. (2011). Pharmacogenetics: From bench to byte—An update of guidelines. Clin. Pharmacol. Ther..

[34] Turner R.M., Newman W.G., Bramon E., McNamee C.J., Wong W.L., Misbah S., Hill S., Caulfield M., Pirmohamed M. (2020). Pharmacogenomics in the UK National Health Service: Opportunities and challenges. Pharmacogenomics.

[35] van Westrhenen R., Aitchison K.J., Ingelman-Sundberg M., Jukić M.M. (2020). Pharmacogenomics of Antidepressant and Antipsychotic Treatment: How Far Have We Got and Where Are We Going?. Front. Psychiatry.

[36] Bycroft C., Freeman C., Petkova D., Band G., Elliott L.T., Sharp K., Motyer A., Vukcevic D., Delaneau O., O’Connell J. (2018). The UK Biobank resource with deep phenotyping and genomic data. Nature.

[37] Sudlow C., Gallacher J., Allen N., Beral V., Burton P., Danesh J., Downey P., Elliott P., Green J., Landray M. (2015). UK Biobank: An Open Access Resource for Identifying the Causes of a Wide Range of Complex Diseases of Middle and Old Age. PLoS Med..

[38] Conomos M.P., Reiner A.P., Weir B.S., Thornton T.A. (2016). Model-free Estimation of Recent Genetic Relatedness. Am. J. Hum. Genet..

[39] Conomos M.P., Miller M.B., Thornton T.A. (2015). Robust inference of population structure for ancestry prediction and correction of stratification in the presence of relatedness. Genet. Epidemiol..

[40] Manichaikul A., Mychaleckyj J.C., Rich S.S., Daly K., Sale M., Chen W.-M. (2010). Robust relationship inference in genome-wide association studies. Bioinformatics.

[41] Gogarten S.M., Bhangale T., Conomos M.P., Laurie C.A., McHugh C.P., Painter I., Zheng X., Crosslin D.R., Levine D., Lumley T. (2012). GWASTools: An R/Bioconductor package for quality control and analysis of genome-wide association studies. Bioinformatics.

[42] Morris J.A., Randall J.C., Maller J.B., Barrett J.C. (2010). Evoker: A visualization tool for genotype intensity data. Bioinformatics.

[43] Browning B., Zhou Y., Browning S. (2016). A one-penny imputed genome from next generation reference panels. Am. J. Hum. Genet..

[44] Browning S.R., Browning B.L. (2007). Rapid and accurate haplotype phasing and missing-data inference for whole-genome association studies by use of localized haplotype clustering. Am. J. Hum. Genet..

[45] Delaneau O., Marchini J.L., McVeanh G.A., Donnelly P., Lunter G., Marchini J.L., Myers S., Gupta-Hinch A., Iqbal Z., Mathieson I. (2014). Integrating sequence and array data to create an improved 1000 Genomes Project haplotype reference panel. Nat. Commun..

[46] Pharmacogene Variation Consortium (PharmVar), (Gaedigk et al. 2018, CPT 103:399; Gaedigk et al. 2019, CPT 105:29. www.PharmVar.org.

[47] Gaedigk A., Simon S.D., Pearce R.E., Bradford L.D., Kennedy M.J., Leeder J.S. (2008). The CYP2D6 activity score: Translating genotype information into a qualitative measure of phenotype. Clin. Pharmacol. Ther..

[48] Gaedigk A., Dinh J.C., Jeong H., Prasad B., Leeder J.S. (2018). Ten years’ experience with the CYP2D6 activity score: A perspective on future investigations to improve clinical predictions for precision therapeutics. J. Pers. Med..

[49] Roopan S., Larsen E.R. (2017). Use of antidepressants in patients with depression and comorbid diabetes mellitus: A systematic review. Acta Neuropsychiatr..

[50] Stingl J., Oesterheld J., Turpeinen M., Lyubimov A.V. (2012). Metabolism of Psychotropic drugs. Encyclopedia of Drug Metabolism and Interaction.

[51] Perneger T.V. (1998). What’s wrong with Bonferroni adjustments. BMJ.

[52] Li M.X., Yeung J.M.Y., Cherny S.S., Sham P.C. (2012). Evaluating the effective numbers of independent tests and significant p-value thresholds in commercial genotyping arrays and public imputation reference datasets. Hum. Genet..

[53] R Core Team. R: (2019). A Language and Environment for Statistical Computing.

[54] Wickham H. (2016). ggplot2: Elegant Graphics for Data Analysis Media.

[55] Dowle M., Srinivasan A. (2019). data.table: Extension of data.frame. https://CRAN.R-project.org/package=data.table.

[56] Zhang J.P., Lencz T., Zhang R.X., Nitta M., Maayan L., John M., Robinson D.G., Fleischhacker W.W., Kahn R.S., Ophoff R.A. (2016). Pharmacogenetic Associations of Antipsychotic Drug-Related Weight Gain: A Systematic Review and Meta-analysis. Schizophr. Bull..

[57] Kumar Y., Kung S., Shinozaki G. (2014). CYP2C19 variation, not citalopram dose nor serum level, is associated with QTc prolongation. J. Psychopharmacol..

[58] Tay J.K.X., Tan C.H., Chong S.A., Tan E.C. (2007). Functional polymorphisms of the cytochrome P450 1A2 (CYP1A2) gene and prolonged QTc interval in schizophrenia. Prog. Neuropsychopharmacol. Biol. Psychiatry.

[59] Lane H.-Y., Liu Y.-C., Huang C.-L., Chang Y.-C., Wu P.-L., Lu C.-T., Chang W.-H. (2006). Risperidone-related weight gain: Genetic and nongenetic predictors. J. Clin. Psychopharmacol..

[60] Correia C.T., Almeida J.P., Santos P.E., Sequeira A.F., Marques C.E., Miguel T.S., Abreu R.L., Oliveira G.G., Vicente A.M. (2010). Pharmacogenetics of risperidone therapy in autism: Association analysis of eight candidate genes with drug efficacy and adverse drug reactions. Pharm. J..

[61] Sukasem C., Hongkaew Y., Ngamsamut N., Puangpetch A., Vanwong N., Chamnanphon M., Chamkrachchangpada B., Sinrachatanant A., Limsila P. (2016). Impact of Pharmacogenetic Markers of CYP2D6 and DRD2 on Prolactin Response in Risperidone-Treated Thai Children and Adolescents with Autism Spectrum Disorders. J. Clin. Psychopharmacol..

[62] Fleeman N., Dundar Y., Dickson R., Jorgensen A., Pushpakom S., McLeod C., Pirmohamed M., Walley T. (2011). Cytochrome P450 testing for prescribing antipsychotics in adults with schizophrenia: Systematic review and meta-analyses. Pharm. J..

[63] Calafato M.S., Austin-Zimmerman I., Thygesen J.H., Sairam M., Metastasio A., Marston L., Abad-Santos F., Bhat A., Harju-Seppänen J., Irizar H. (2020). The effect of CYP2D6 variation on antipsychotic-induced hyperprolactinaemia: A systematic review and meta-analysis. Pharm. J..

[64] Chávez-Castillo M., Ortega Á., Nava M., Fuenmayor J., Lameda V., Velasco M., Bermúdez V., Rojas-Quintero J. (2018). Metabolic Risk in Depression and Treatment with Selective Serotonin Reuptake Inhibitors: Are the Metabolic Syndrome and an Increase in Cardiovascular Risk Unavoidable?. Vessel Plus..

[65] Pan A., Sun Q., Okereke O.I., Rexrode K.M., Rubin R.R., Lucas M., Willett W.C., Manson J.E., Hu F.B. (2012). Use of antidepressant medication and risk of type 2 diabetes: Results from three cohorts of US adults. Diabetologia.

[66] Yoon J.M., Cho E.-G., Lee H.-K., Park S.M. (2013). Antidepressant use and diabetes mellitus risk: A meta-analysis. Korean J. Fam. Med..

[67] Heald A.H., Stedman M., Davies M., Livingston M., Taylor D., Gadsby R. (2020). Antidepressant Prescribing in England: Patterns and Costs. Prim. Care Companion CNS Disord..

[68] Sindrup S.H., Brøsen K., Gram L.F., Hallas J., Skjelbo E., Allen A., Allen G.D., Cooper S.M., Mellows G., Tasker T.C. (1992). The relationship between paroxetine and the sparteine oxidation polymorphism. Clin. Pharmacol. Ther..

[69] Solai L.K., Pollock B.G., Mulsant B.H., Frye R.F., Miller M.D., Sweet R.A., Kirshner M., Sorisio D., Begley A., Reynolds C.F. (2002). Effect of nortriptyline and paroxetine on CYP2D6 activity in depressed elderly patients. J. Clin. Psychopharmacol..

[70] Zourková A., Hadasová E. (2003). Paroxetine-induced conversion of cytochrome P450 2D6 phenotype and occurence of adverse effects. Gen. Physiol. Biophys..

[71] Khoza S., Barner J.C., Bohman T.M., Rascati K., Lawson K., Wilson J.P. (2012). Use of antidepressant agents and the risk of type 2 diabetes. Eur. J. Clin. Pharmacol..

[72] Biagetti B., Corcoy R. (2013). Hypoglycemia associated with fluoxetine treatment in a patient with type 1 diabetes. World J. Clin. Cases.

[73] Rebai R., Jasmin L., Boudah A. (2017). The antidepressant effect of melatonin and fluoxetine in diabetic rats is associated with a reduction of the oxidative stress in the prefrontal and hippocampal cortices. Brain Res. Bull..

[74] Baumeister H., Hutter N., Bengel J. (2012). Psychological and Pharmacological Interventions for Depression in Patients with Diabetes Mellitus and Depression. Cochrane Database Syst. Rev..

[75] Baumann P., Jonzier-Perey M., Koeb L., Küpfer A., Tinguely D., Schöpf J. (1986). Amitriptyline pharmacokinetics and clinical response: II. Metabolic polymorphism assessed by hydroxylation of debrisoquine and mephenytoin. Int. Clin. Psychopharmacol..

[76] Dean L., Pratt V.M., Scott S.A., Pirmohamed M., Esquivel B., Kane M.S., Kattman B.L., Malheiro A.J. (2012). Amitriptyline Therapy and CYP2D6 and CYP2C19 Genotype. Medical Genetics Summaries.

[77] Gaziano J.M., Concato J., Brophy M., Fiore L., Pyarajan S., Breeling J., Whitbourne S., Deen J., Shannon C., Humphries D. (2016). Million Veteran Program: A mega-biobank to study genetic influences on health and disease. J. Clin. Epidemiol..

[78] Rehman A., Setter S.M., Vue M.H. (2011). Drug-induced glucose alteraions part 2: Drug-induced hyperglycemia. Diabetes Spectr..

[79] Xue A., Wu Y., Zhu Z., Zhang F., Kemper K.E., Zheng Z., Yengo L., Lloyd-Jones L.R., Sidorenko J.S., Wu Y. (2018). Genome-wide association analyses identify 143 risk variants and putative regulatory mechanisms for type 2 diabetes. Nat. Commun..

[80] Zhou Y., Ingelman-Sundberg M., Lauschke V.M. (2017). Worldwide Distribution of Cytochrome P450 Alleles: A Meta-analysis of Population-scale Sequencing Projects. Clin. Pharmacol. Ther..

[81] McInnes G., Lavertu A., Sangkuhl K., Klein T.E., Whirl-Carrillo M., Altman R.B. (2020). Pharmacogenetics at Scale: An Analysis of the UK Biobank. Clin. Pharmacol. Ther..

[82] Müller D.J., Kekin I., Kao A.C.C., Brandl E.J. (2013). Towards the implementation of CYP2D6 and CYP2C19 genotypes in clinical practice: Update and report from a pharmacogenetic service clinic. Int. Rev. Psychiatry Abingdon Engl..

[83] Altar C.A., Carhart J.M., Allen J.D., Hall-Flavin D.K., Dechairo B.M., Winner J.G. (2015). Clinical validity: Combinatorial pharmacogenomics predicts antidepressant responses and healthcare utilizations better than single gene phenotypes. Pharm. J..

[84] Arranz M.J., Gonzalez-Rodriguez A., Perez-Blanco J., Penadés R., Gutierrez B., Ibañez L., Arias B., Brunet M., Cervilla J., Salazar J. (2019). A pharmacogenetic intervention for the improvement of the safety profile of antipsychotic treatments. Transl. Psychiatry.

[85] Greden J.F., Parikh S.V., Rothschild A.J., Thase M.E., Dunlop B.W., DeBattista C., Conway C.R., Forester B.P., Mondimore F.M., Shelton R.C. (2019). Impact of pharmacogenomics on clinical outcomes in major depressive disorder in the GUIDED trial: A large, patient- and rater-blinded, randomized, controlled study. J. Psychiatr. Res..

[86] Herbild L., Andersen S.E., Werge T., Rasmussen H.B., Jürgens G. (2013). Does pharmacogenetic testing for CYP450 2D6 and 2C19 among patients with diagnoses within the schizophrenic spectrum reduce treatment costs?. Basic Clin. Pharmacol. Toxicol..

