# Supplementary material for: The Influence of CYP2D6 and CYP2C19 Genetic Variation on Diabetes Mellitus Risk in People Taking Antidepressants and Antipsychotics
Source: Genes (Basel). 2021 Nov 3;12(11):1758. doi: 10.3390/genes12111758 (PMC8620997; doi:10.3390/genes12111758)
Supplement: Supplementary file 1 [file genes-12-01758-s001.zip › genes-1423997-supplementary.pdf]

Supplementary material

Methodology: Sample

The medication data recorded in the UK Biobank was collected through a verbal interview with a trained nurse. Participants were asked if they were currently taking any regular prescription medication. If they answered yes, the nurse asked them to detail medication they were taking. Dose, formulation or duration of treatment were not recorded, and any short-term medication (i.e., one week course of antibiotics) were not included.

A psychotropic drug was defined as any drug indicated, either officially licenced or through routine clinical practice, for the treatment of psychosis (schizophrenia), depression or bipolar disorder. Anxiolytic agents were not included in this study. The drugs of interest were identified through review of the British National Formulary (BNF) Drug Dictionary[1]. The list was expanded to include drugs licenced overseas and drugs no longer listed in the BNF. As the medication data in the UK Biobank are self-reported, it was necessary to identify all potential names (brand names and generic names in foreign languages) of the medications of interest. These alternate names were identified through review of published drug labels, the websites of the European Medicines Agency (EMA) [2], the Food and Drug Administration (FDA)[3], the Mayo Clinic[4], the independent website www.drugs.com[5] and the National Library of Medicine[6].

---

[1] MedicinesComplete - British National Formulary. https://www.medicinescomplete.com/mc/bnf/current/
[2] European Medicines Agency. https://www.ema.europa.eu/
[3] U S Food and Drug Administration. https://www.fda.gov/
[4] Mayo Clinic. https://www.mayoclinic.org/
[5] Drugs.com | Prescription Drug Information, Interactions & Side Effects. https://www.drugs.com/
[6] National Library of Medicine’s LactMed Database. https://toxnet.nlm.nih.gov/newtoxnet/lactmed.htm
[7] Adapted from: CONSORT 2010 Statement, BMJ 2010;340:c332 http://www.consort-statement.org/

Supplementary Figure S1. Adapted CONSORT 2010 statement[7]

CONSORT
TRANSPARENT REPORTING of TRIALS

CONSORT 2010 Flow Diagram

Enrollment

Assessed for eligibility (n= 502,604)

Excluded

Excluded total (n= 469,455)

- Withdrew consent[a] (n= 61)
- Not taking psychotropic drug of interest (n= 458,492)
- No HbA1c measurement (n= 3,268)
- Inconsistent diabetes status (n= 49)
- Missing genetic data or failed genetic sample level quality control (n= 7,250)
- Relatedness (n= 335)

Allocation

Included in analyses (n= 33,149)

- Takes antidepressant (n= 31,579)[b]
- Takes antipsychotic (n= 2,999)[b]

Assigned CYP phenotype

CYP2D6

- Ultra-rapid metaboliser: N/A[c]
- Normal metaboliser: n= 23,585
- Intermediate metaboliser: n= 7,824
- Poor metaboliser: n= 1,740

CYP2C19

- Ultra-rapid metaboliser: n= 1,411
- Rapid metabolisers: n= 8,193
- Normal metaboliser: n= 12,592
- Intermediate metaboliser: n= 9,825
- Poor metaboliser: n= 1,128

[a] Number of participants who withdrew consent after the initial download of all phenotype data and before the analysis was conducted. Participants who withdrew consent prior to initial download of data were already removed; [b] Note that several subjects report taking both antidepressant(s) and antipsychotic(s) and are included in both analyses; [c] See main body of text for detail on why CYP2D6 ultra-rapid metabolisers were not defined in this analysis.

Methodology: Covariates

Diabetes

There were several items in the UK Biobank which could act as a source of information for whether the patient had diabetes or not, namely ICD-10 diagnosis, self-reported diagnosis of diabetes or self-reported use of antidiabetic medications. Though this would be the most reliable item to use for the purpose of our research, the ICD-10 data was incomplete, covering 410,316 participants and recording 3399 (0.82%) cases of diabetes mellitus. Based on self-reported data, the prevalence of diabetes mellitus in the UK Biobank was 5.28%. This is consistent with UK epidemiological studies which report the prevalence of diabetes mellitus at 7%[7]. We thus concluded that the self-reported data was more reliable in this instance. Based on the data available we were unable to differentiate between type 1 and type 2 diabetes.

Anti-diabetic medications

We identified participants taking insulin, metformin, thiazolidinediones, sulfonylureas, meglitinides, alpha-glucosidase inhibitors. There were no participants within our dataset who have been taking glucagon-like peptide-1 or gastric inhibitory peptide as well as gliflozins. We also identified all brand names of anti-diabetic medications and converted these to their generic equivalents and created a dichotomous variable which reflected the use of the antidiabetic medications.

BMI

A high BMI is an independent risk factor for diabetes; hence it was included in our analyses. BMI at baseline was downloaded directly from the UK Biobank.

Enzyme inhibitors

We identified any participants taking drugs that inhibit CYP2D6 and CYP2C19 activity[8]. In the sample, 1,969 participants were taking a CYP2D6 inhibitor drug including: ranitidine, celecoxib, metoclopramide, chlorphenamine, terbinafine, hydroxyzine or promethazine. We also identified 8,340 participants taking drugs that are known CYP2C19 inhibitors including omeprazole, esomeprazole, lansoprazole, pantoprazole, oestrogen, cimetidine, modafinil, topiramate, indomethacin or oxcarbazepine.

---

[7] Whicher, C. A., O'Neill, S., & Holt, R. I. G. (2020). Diabetes in the UK: 2019. *Diabetic Medicine*, *37* (2), 242–247. https://doi.org/10.1111/dme.14225

[8] *CYTOCHROME P450 DRUG INTERACTION TABLE - Drug Interactions*. (n.d.). Retrieved September 1, 2020, from https://drug-interactions.medicine.iu.edu/MainTable.asp

Covariates in antidepressants' model

As expected, in every antidepressants' model, having diabetes, taking antidiabetic medications, raising BMI and increasing age was associated with higher HbA1c (all p <0.001). In all models apart from venlafaxine, South Asian ethnicity was associated with higher HbA1c level (p range <0.001 - 0.050). African ethnicity was associated with higher HbA1c in following models: citalopram, paroxetine, venlafaxine (p range <0.001 – 0.005). Admix Caucasian ethnicity was associated with higher HbA1c levels in fluoxetine models (p = 0.006 and p = 0.027 accordingly) and Other ethnicity in tricyclic and amitriptyline models (p <0.001, p = 0.002). Male sex was associated with higher HbA1c in citalopram and fluoxetine models. Please refer to supplementary tables 10, 11, 14, 16, 17, 18, 21.

Methods: Assigning metabolic phenotype

We extracted regions of interest for each CYP2D6 and CYP2C19, defined as being 1 megabase (Mb) either side of each gene. Start and stop coordinates are provided in supplementary table 1 and were identified using the University of California Santa Cruz Human Genome Browser[9].

Supplementary Table S1. Showing start and stop positions used to extract relevant genetic data from full UKB sample.

GENE (CHROMOSOME, POSITION)	START POSITION FOR DATA EXTRACTION	STOP POSITION FOR DATA EXTRACTION
**CYP2C19 (CHR10: 96447882- 96612671)**	95447882	97612671
**CYP2D6 (CHR 22: 42522501- 42526883)**	41522501	43526883

Supplementary Table S2. Frequencies of CYP2C19 diplotypes and metabolic phenotypes in 33,149 Biobank participants taking antidepressants or antipsychotics.

Diplotype	Frequency (N)	Percentage (%)
**Poor metaboliser**		
CYP2C19*1 / CYP2C19*1	10270	30.98
CYP2C19*1 / CYP2C19*13	1080	3.26
CYP2C19*13 / CYP2C19*1	1034	3.12
CYP2C19*13 / CYP2C19*13	104	0.31
**Intermediate metaboliser**		

[9] Kent W, Sugnet C, Furey T, et al. The Human Genome Browser at UCSC. *Genome Res*. 2002;12 (6):996-1006.

CYP2C19*1 / CYP2C19*2	2843	8.58
CYP2C19*2 / CYP2C19*1	2766	8.34
CYP2C19*17 / CYP2C19*2	1048	3.16
CYP2C19*2 / CYP2C19*17	969	2.92
CYP2C19*1 / CYP2C19*3	475	1.43
CYP2C19*3 / CYP2C19*1	448	1.35
CYP2C19*13 / CYP2C19*2	333	1
CYP2C19*2 / CYP2C19*13	289	0.87
CYP2C19*17 / CYP2C19*3	183	0.55
CYP2C19*3 / CYP2C19*17	166	0.5
**Normal metaboliser**		
CYP2C19*2 / CYP2C19*2	773	2.33
CYP2C19*3 / CYP2C19*2	152	0.46
CYP2C19*2 / CYP2C19*3	125	0.38
**Rapid metaboliser**		
CYP2C19*1 / CYP2C19*17	3755	11.33
CYP2C19*17 / CYP2C19*1	3667	11.06
CYP2C19*13 / CYP2C19*17	382	1.15
CYP2C19*17 / CYP2C19*13	357	01.08
**Ultra-rapid metaboliser**		
CYP2C19*17 / CYP2C19*17	1411	4.26

‘/’ distinguishes two homologous chromosomes. ‘;’ connects several different alleles on the same chromosome

Supplementary Table S3. Frequencies of CYP2D6 diplotypes and metabolic phenotypes in 33,149 Biobank participants taking antidepressants or antipsychotics.

Diplotype	Frequency (N)	Percentage (%)
**Normal metaboliser**		

CYP2D6*29 / CYP2D6*29	5716	17.24
CYP2D6*1 / CYP2D6*29	2848	8.59
CYP2D6*29 / CYP2D6*1	2751	8.3
CYP2D6*1 / CYP2D6*1	2049	6.18
CYP2D6*4 / CYP2D6*1	1556	4.69
CYP2D6*1 / CYP2D6*4	1533	4.62
CYP2D6*29 / CYP2D6*41	1157	3.49
CYP2D6*41 / CYP2D6*29	1155	3.48
CYP2D6*41 / CYP2D6*1	853	2.57
CYP2D6*1 / CYP2D6*41	815	2.46
CYP2D6*29 / CYP2D6*9;CYP2D6*29	416	1.25
CYP2D6*9;CYP2D6*29 / CYP2D6*29	408	1.23
CYP2D6*41 / CYP2D6*41	337	1.02
CYP2D6*1 / CYP2D6*9;CYP2D6*29	208	0.63
CYP2D6*9;CYP2D6*29 / CYP2D6*1	186	0.56
CYP2D6*10 / CYP2D6*29	158	0.48
CYP2D6*3A / CYP2D6*1	132	0.4
CYP2D6*29 / CYP2D6*10	129	0.39
CYP2D6*1 / CYP2D6*3A	118	0.36
**Intermediate metaboliser**		
CYP2D6*4 / CYP2D6*29	2512	7.58
CYP2D6*29 / CYP2D6*4	2331	7.03
CYP2D6*41 / CYP2D6*4	611	1.84
CYP2D6*4 / CYP2D6*41	589	1.78
CYP2D6*29 / CYP2D6*3A	246	0.74
CYP2D6*3A / CYP2D6*29	244	0.74

CYP2D6*10 / CYP2D6*4	222	0.67
CYP2D6*4 / CYP2D6*10	219	0.66
CYP2D6*4 / CYP2D6*9;CYP2D6*29	169	0.51
CYP2D6*9;CYP2D6*29 / CYP2D6*4	140	0.42
**Poor metaboliser**		
CYP2D6*4 / CYP2D6*4	1468	4.43
CYP2D6*3A / CYP2D6*4	103	0.31
CYP2D6*4 / CYP2D6*3A	95	0.29

'/' distinguishes two homologous chromosomes. ';' connects several different alleles on the same chromosome

Supplementary Table S4. HbA1c levels and CYP phenotypes across individual and groups of medications.

	**CYP2D6**			**CYP2C19**				
	PM	IM	NM	PM	IM	NM	RM	UM
**Models**	HbA1c *[mmol/mol]**, (SD)							
Antipsychotics	36.78 (7.26)	37.60 (8.54)	37.53 (8.30)	-	-	-	-	-
Tricyclics	37.60 (7.79)	37.79 (8.38)	37.86 (8.21)	37.75 (7.80)	37.6 (7.73)	37.88 (8.30)	38.00 (8.81)	38.13 (7.88)
Amitriptyline	37.52 (7.86)	37.93 (8.60)	37.85 (8.18)	37.61 (7.28)	37.64 (7.84)	37.92 (8.31)	38.00 (8.86)	38.08 (8.06)
Fluoxetine	36.50 (6.90)	36.47 (6.50)	36.65 (7.50)	-	-	-	-	-
Paroxetine	40.46 (15.05)	37.50 (8.27)	37.38 (7.27)	-	-	-	-	-
Citalopram	-	-	-	36.89 (7.54)	36.48 (6.90)	36.67 (7.56)	36.51 (7.23)	36.01 (6.16)
Sertraline	-	-	-	35.30 (4.35)	36.99 (7.24)	37.15 (7.43)	37.13 (7.37)	37.04 (8.50)

Venlafaxine	39.49 (12.38)	37.13 (8.56)	37.58 (8.06)	-	-	-	-	-

* HbA1c levels diagnostic for impaired glucose regulation: normal < 42 mmol/mol, prediabetes 42 - 47 mmol/mol, diabetes ≥48 mmol/mol

Supplementary Table S5. Characteristics of CYP2C19 metabolic phenotype in our sample

	NM (N=12592)	IM (N=9825)	PM (N=1128)	RM (N=8193)	UM (N=1411)	Overall (N=33149)
**Age (years)**						
Mean (SD)	56.6 (7.79)	56.6 (7.85)	56.2 (8.01)	56.7 (7.72)	56.6 (7.80)	56.6 (7.80)
**Sex**						
Female	8623 (68.5%)	6751 (68.7%)	749 (66.4%)	5547 (67.7%)	962 (68.2%)	22632 (68.3%)
Male	3969 (31.5%)	3074 (31.3%)	379 (33.6%)	2646 (32.3%)	449 (31.8%)	10517 (31.7%)
**Ethnicity**						
European	11762 (93.4%)	9205 (93.7%)	1062 (94.1%)	7670 (93.6%)	1307 (92.6%)	31006 (93.5%)
Admix European	342 (2.7%)	223 (2.3%)	27 (2.4%)	198 (2.4%)	46 (3.3%)	836 (2.5%)
African	127 (1.0%)	110 (1.1%)	10 (0.9%)	95 (1.2%)	16 (1.1%)	358 (1.1%)
East Asian	25 (0.2%)	13 (0.1%)	1 (0.1%)	12 (0.1%)	0 (0%)	51 (0.2%)
Other	178 (1.4%)	149 (1.5%)	19 (1.7%)	109 (1.3%)	24 (1.7%)	479 (1.4%)
South Asian	158 (1.3%)	125 (1.3%)	9 (0.8%)	109 (1.3%)	18 (1.3%)	419 (1.3%)
**HbA1c (mmol/mol)**						
Mean (SD)	37.2 (7.89)	37.0 (7.44)	37.0 (7.02)	37.2 (7.90)	37.1 (7.84)	37.1 (7.73)
**Diabetes**						
Yes	1117 (8.9%)	827 (8.4%)	105 (9.3%)	762 (9.3%)	125 (8.9%)	2936 (8.9%)
No	11475 (91.1%)	8998 (91.6%)	1023 (90.7%)	7431 (90.7%)	1286 (91.1%)	30213 (91.1%)
**Taking antidiabetic medication**						
Yes	819 (6.5%)	580 (5.9%)	67 (5.9%)	532 (6.5%)	100 (7.1%)	2098 (6.3%)

No	11773 (93.5%)	9245 (94.1%)	1061 (94.1%)	7661 (93.5%)	1311 (92.9%)	31051 (93.7%)
**BMI**						
Mean (SD)	28.8 (5.67)	28.7 (5.69)	28.6 (5.34)	28.7 (5.66)	28.6 (5.73)	28.7 (5.67)
**Taking CYP2C19 inhibitor**						
Yes	3184 (25.3%)	2364 (24.1%)	283 (25.1%)	2068 (25.2%)	360 (25.5%)	8259 (24.9%)
No	9408 (74.7%)	7461 (75.9%)	845 (74.9%)	6125 (74.8%)	1051 (74.5%)	24890 (75.1%)

Supplementary Table S6. Characteristics of CYP2D6 metabolic phenotype in our sample

	NM (N=23585)	IM (N=7824)	PM (N=1740)	Overall (N=33149)
**Age (years)**				
Mean (SD)	56.6 (7.80)	56.6 (7.82)	56.6 (7.71)	56.6 (7.80)
Median [Min, Max]	58.0 [40.0, 71.0]	58.0 [40.0, 70.0]	58.0 [40.0, 70.0]	58.0 [40.0, 71.0]
**Sex**				
Female	16086 (68.2%)	5355 (68.4%)	1191 (68.4%)	22632 (68.3%)
Male	7499 (31.8%)	2469 (31.6%)	549 (31.6%)	10517 (31.7%)
**Ethnicity**				
Caucasian	22027 (93.4%)	7342 (93.8%)	1637 (94.1%)	31006 (93.5%)
Admix Caucasian	620 (2.6%)	181 (2.3%)	35 (2.0%)	836 (2.5%)
African	262 (1.1%)	78 (1.0%)	18 (1.0%)	358 (1.1%)
East Asian	36 (0.2%)	10 (0.1%)	5 (0.3%)	51 (0.2%)
Other	346 (1.5%)	108 (1.4%)	25 (1.4%)	479 (1.4%)
South Asian	294 (1.2%)	105 (1.3%)	20 (1.1%)	419 (1.3%)
**HbA1c (mmol/mol)**				
Mean (SD)	37.1 (7.74)	37.0 (7.57)	37.3 (8.34)	37.1 (7.73)
**Diabetes**				
Yes	2106 (8.9%)	670 (8.6%)	160 (9.2%)	2936 (8.9%)
No	21479 (91.1%)	7154 (91.4%)	1580 (90.8%)	30213 (91.1%)
**Taking antidiabetic medication**				

Yes	1488 (6.3%)	487 (6.2%)	123 (7.1%)	2098 (6.3%)
No	22097 (93.7%)	7337 (93.8%)	1617 (92.9%)	31051 (93.7%)
**BMI (kg/m²)**				
Mean (SD)	28.7 (5.65)	28.8 (5.67)	28.8 (5.80)	28.7 (5.67)
**Taking CYP2D6 inhibitor**				
Yes	1401 (5.9%)	440 (5.6%)	102 (5.9%)	1943 (5.9%)
No	22184 (94.1%)	7384 (94.4%)	1638 (94.1%)	31206 (94.1%)

Supplementary Table S7. CYP2D6 metabolic phenotypes of people taking antidepressants

	NM (N=23749)	IM (N=7816)	PM (N=1757)	Overall (N=33367)
Antidepressant	23794 (100%)	7816 (100%)	1757 (100%)	33367 (100%)
**Tricyclic**				
amitriptyline	5840 (24.5%)	1929 (24.7%)	422 (24.0%)	8191 (24.5%)
dosulepin	1158 (4.9%)	349 (4.5%)	64 (3.6%)	1571 (4.7%)
lofepramine	231 (1.0%)	71 (0.9%)	21 (1.2%)	323 (1.0%)
clomipramine	221 (0.9%)	91 (1.2%)	8 (0.5%)	320 (1.0%)
nortriptyline	197 (0.8%)	63 (0.8%)	12 (0.7%)	272 (0.8%)
imipramine	139 (0.6%)	51 (0.7%)	15 (0.9%)	205 (0.6%)
trimipramine	62 (0.3%)	19 (0.2%)	4 (0.2%)	85 (0.3%)
**SSRI**				
citalopram	5381 (22.6%)	1753 (22.4%)	411 (23.4%)	7545 (22.6%)
fluoxetine	3888 (16.3%)	1282 (16.4%)	299 (17.0%)	5469 (16.4%)
sertraline	1394 (5.9%)	456 (5.8%)	105 (6.0%)	1955 (5.9%)
paroxetine	1367 (5.7%)	457 (5.8%)	106 (6.0%)	1930 (5.8%)
escitalopram	795 (3.3%)	249 (3.2%)	55 (3.1%)	1099 (3.3%)
**SNRI**				
venlafaxine	1354 (5.7%)	430 (5.5%)	103 (5.9%)	1887 (5.7%)
duloxetine	325 (1.4%)	119 (1.5%)	16 (0.9%)	460 (1.4%)
**Tetracyclic**				
mirtazapine	869 (3.7%)	316 (4.0%)	74 (4.2%)	1259 (3.8%)

**SARI**				
trazodone	412 (1.7%)	139 (1.8%)	33 (1.9%)	584 (1.8%)
**NRI**				
reboxetine	38 (0.2%)	10 (0.1%)	6 (0.3%)	54 (0.2%)
**OTHER**				
other	123 (0.5%)	32 (0.4%)	3 (0.2%)	158 (0.5%)

NM - normal metaboliser, PM - poor metaboliser, IM – intermediate metaboliser, SSRI – Selective Serotonin Reuptake Inhibitor, SNRI – Selective Noradrenaline Reuptake Inhibitor, MOI – Monoamine Oxidase Inhibitor, NRI – Noradrenaline Reuptake Inhibitor, NDRI – Noradrenaline Dopamine Reuptake Inhibitor, SARI – Serotonin Antagonist and Reuptake Inhibitor

Other - doxepin, fluvoxamine, phenelzine, moclobemide, tranylcypromine, bupropion, mianserin, isocarboxazid

Supplementary Table S8. CYP2C19 metabolic phenotypes of people taking antidepressants

	NM (N=12689)	IM (N=9889)	PM (N=1122)	RM (N=8241)	UM (N=1426)	Overall (N=33367)
Antidepressant	12689 (100%)	9889 (100%)	1122 (100%)	8241 (100%)	1426 (100%)	33367 (100%)
**Tricyclic**						
amitriptyline	3116 (24.6%)	2483 (25.1%)	263 (23.4%)	1961 (23.8%)	368 (25.8%)	8191 (24.5%)
dosulepin	603 (4.8%)	439 (4.4%)	60 (5.3%)	388 (4.7%)	81 (5.7%)	1571 (4.7%)
lofepramine	127 (1.0%)	95 (1.0%)	4 (0.4%)	82 (1.0%)	15 (1.1%)	323 (1.0%)
clomipramine	126 (1.0%)	96 (1.0%)	12 (1.1%)	75 (0.9%)	11 (0.8%)	320 (1.0%)
nortriptyline	98 (0.8%)	73 (0.7%)	7 (0.6%)	85 (1.0%)	9 (0.6%)	272 (0.8%)
imipramine	74 (0.6%)	59 (0.6%)	5 (0.4%)	59 (0.7%)	8 (0.6%)	205 (0.6%)
trimipramine	38 (0.3%)	26 (0.3%)	2 (0.2%)	13 (0.2%)	6 (0.4%)	85 (0.3%)
**SSRI**						
citalopram	2923 (23.0%)	2205 (22.3%)	232 (20.7%)	1882 (22.8%)	303 (21.2%)	7545 (22.6%)
fluoxetine	2065 (16.3%)	1642 (16.6%)	195 (17.4%)	1338 (16.2%)	229 (16.1%)	5469 (16.4%)
sertraline	760 (6.0%)	587 (5.9%)	67 (6.0%)	465 (5.6%)	76 (5.3%)	1955 (5.9%)
paroxetine	731 (5.8%)	580 (5.9%)	79 (7.0%)	478 (5.8%)	62 (4.3%)	1930 (5.8%)

escitalopram	396 (3.1%)	345 (3.5%)	39 (3.5%)	263 (3.2%)	56 (3.9%)	1099 (3.3%)
**SNRI**						
venlafaxine	717 (5.7%)	536 (5.4%)	71 (6.3%)	483 (5.9%)	80 (5.6%)	1887 (5.7%)
duloxetine	167 (1.3%)	143 (1.4%)	20 (1.8%)	105 (1.3%)	25 (1.8%)	460 (1.4%)
**Tetracyclic**						
mirtazapine	439 (3.5%)	359 (3.6%)	40 (3.6%)	356 (4.3%)	65 (4.6%)	1259 (3.8%)
**SARI**						
trazodone	220 (1.7%)	169 (1.7%)	18 (1.6%)	155 (1.9%)	22 (1.5%)	584 (1.8%)
**NRI**						
reboxetine	21 (0.2%)	15 (0.2%)	2 (0.2%)	13 (0.2%)	3 (0.2%)	54 (0.2%)
**OTHER**						
other	68 (0.5%)	37 (0.4%)	6 (0.5%)	40 (0.5%)	7 (0.5%)	158 (0.5%)

NM - normal metaboliser, PM - poor metaboliser, IM – intermediate metaboliser, RM – rapid metaboliser, UM – ultra-rapid metaboliser, SSRI – Selective Serotonin Reuptake Inhibitor, SNRI – Selective Noradrenaline Reuptake Inhibitor, MOI – Monoamine Oxidase Inhibitor, NRI – Noradrenaline Reuptake Inhibitor, NDRI – Noradrenaline Dopamine Reuptake Inhibitor, SARI – Serotonin Antagonist and Reuptake Inhibitor, Other - doxepin, fluvoxamine, phenelzine, moclobemide, tranylcypromine, bupropion, mianserin, isocarboxazid

Supplementary Table S9. CYP2D6 metabolic phenotype of individuals taking antipsychotics

	**NM** (N=2004)	**IM** (N=671)	**PM** (N=142)	**Overall** (N=2817)
Antipsychotic	2004 (100%)	671 (100%)	142 (100%)	2817 (100%)
**Medication**				
prochlorperazine	607 (30.3%)	221 (32.9%)	42 (29.6%)	870 (30.9%)
olanzapine	352 (17.6%)	114 (17.0%)	33 (23.2%)	499 (17.7%)
quetiapine	215 (10.7%)	74 (11.0%)	12 (8.5%)	301 (10.7%)
risperidone	181 (9.0%)	56 (8.3%)	10 (7.0%)	247 (8.8%)
chlorpromazine	105 (5.2%)	40 (6.0%)	6 (4.2%)	151 (5.4%)
flupentixol	107 (5.3%)	36 (5.4%)	5 (3.5%)	148 (5.3%)
trifluoperazine	110 (5.5%)	25 (3.7%)	6 (4.2%)	141 (5.0%)
amisulpride	57 (2.8%)	17 (2.5%)	5 (3.5%)	79 (2.8%)

haloperidol	51 (2.5%)	17 (2.5%)	5 (3.5%)	73 (2.6%)
aripiprazole	43 (2.1%)	16 (2.4%)	4 (2.8%)	63 (2.2%)
sulpiride	41 (2.0%)	14 (2.1%)	4 (2.8%)	59 (2.1%)
other	135 (6.7%)	41 (6.1%)	10 (7.0%)	186 (6.6%)

NM - normal metaboliser, PM - poor metaboliser, IM – intermediate metaboliser. Other - fluphenazine, clozapine, promazine, zuclopenthxol, perphenazine, pipotiazine, periciazine, levomepromazine, benperidol, pimozide, thioridazine, sertindole

Supplementary Figure S2. Interaction between diabetes status and metabolic phenotypes among subjects taking, from left to right, (a) tricyclic antidepressants; (b) Amitriptyline; (c) Fluoxetine; (d) Venlafaxine; (e) Citalopram; (f) Sertraline.

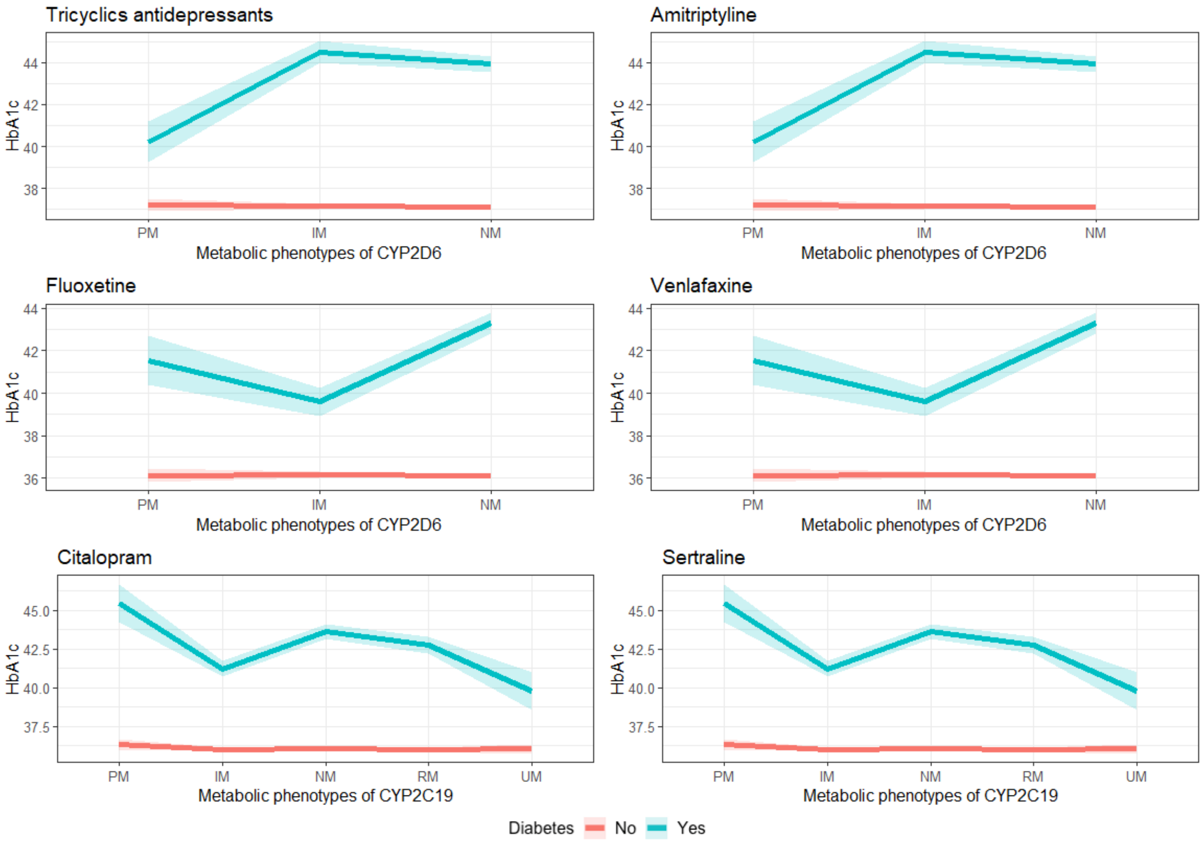

Supplementary Table S10. Association between CYP2D6 metabolic phenotype and HbA1c within individuals taking paroxetine – additional detail

	Paroxetine		
Predictors	*Estimates*	*CI*	*p*
CYP2D6 IM	0.23	-0.42,0.87	0.489
CYP2D6 PM	2.43	1.23,3.63	**<0.001**

Takes CYP2D6 inhibitor	-0.34	-1.55,0.87	0.577
Sex: Male	0.41	-0.16,0.98	0.158
Age at recruitment	0.15	0.11,0.19	**<0.001**
Ethnicity: Admix Caucasian	0.14	-1.39,1.68	0.856
Ethnicity: African	6.41	2.96,9.86	**<0.001**
Ethnicity: East Asian	-0.90	-12.83,11.03	0.882
Ethnicity: Other	0.56	-1.94,3.06	0.661
Ethnicity: South Asian	6.76	3.92,9.59	**<0.001**
Diabetes	6.85	5.11,8.59	**<0.001**
BMI	0.14	0.09,0.19	**<0.001**
Antidiabetics	12.89	10.88,14.89	**<0.001**
Observations	1930		
R^2 / R^2 adjusted	0.454 / 0.450		

Supplementary Table S11. Association between CYP2D6 metabolic phenotype and HbA1c within individuals taking fluoxetine – additional detail

	**Fluoxetine**		
Predictors	*Estimates*	*CI*	*p*
CYP2D6 IM	0.06	-0.29,0.41	0.728
CYP2D6 PM	0.04	-0.62,0.69	0.916
Diabetes: CYP2D6 IM	-3.78	-5.03,-2.53	**<0.001**
Diabetes: CYP2D6 PM	-1.81	-4.11,0.49	0.124
Takes CYP2D6 inhibitor	0.01	-0.62,0.64	0.970
Sex: Male	0.36	0.04,0.67	**0.027**
Age at recruitment	0.15	0.13,0.17	**<0.001**
Ethnicity: Admix Caucasian	1.27	0.36,2.17	**0.006**

Ethnicity: African	0.80	-0.75,2.34	0.314
Ethnicity: East Asian	1.84	-1.63,5.31	0.299
Ethnicity: Other	0.28	-0.91,1.47	0.640
Ethnicity: South Asian	3.68	2.03,5.34	**<0.001**
Diabetes	7.22	6.20,8.23	**<0.001**
BMI	0.16	0.13,0.18	**<0.001**
Antidiabetics	12.50	11.39,13.62	**<0.001**
Observations	5469		
R^2 / R^2 adjusted	0.467 / 0.465		

Supplementary Table S12. Association between CYP2D6 metabolic phenotype and HbA1c within individuals taking venlafaxine – additional detail

	**Venlafaxine**		
Predictors	*Estimates*	*CI*	*p*
CYP2D6 IM	-0.23	-0.89,0.43	0.489
CYP2D6 PM	-0.46	-1.73,0.80	0.495
Diabetes: CYP2D6 IM	3.62	1.27,5.98	**0.003**
Diabetes: CYP2D6 PM	11.44	8.05,14.84	**4.79e-11**
Takes CYP2D6 inhibitor	-0.37	-1.50,0.77	0.525
Sex: Male	0.41	-0.14,0.97	0.146
Age at recruitment	0.14	0.11,0.18	**<0.001**
Ethnicity: Admix Caucasian	0.42	-1.17,2.02	0.602
Ethnicity: African	4.59	1.37,7.80	**0.005**
Ethnicity: East Asian	-0.96	-12.42,10.51	0.870
Ethnicity: Other	1.97	-0.62,4.56	0.136
Ethnicity: South Asian	2.63	-0.09,5.35	0.058

Diabetes	5.68	4.04,7.33	**1.77e-11**
BMI	0.15	0.10,0.20	**<0.001**
Antidiabetics	14.82	12.97,16.67	**<0.001**
Observations	1885		
R^2 / R^2 adjusted	0.528 / 0.524		

Model adjusted by age, ethnicity, sex, taking inhibitors of CYP2D6, taking antidiabetics and BMI

Normal metabolisers of CYP2D6: 1,352; normal metabolisers of CYP2D6:diabetes = 135

Supplementary Table S13. Association between CYP2C19 metabolic phenotype and HbA1c within individuals taking citalopram

		**Citalopram**	
Predictors	*Estimates*	*CI*	*p*
CYP2C19 IM	-0.06	-0.36,0.24	0.701
CYP2C19 PM	0.25	-0.48,0.99	0.500
CYP2C19 RM	-0.07	-0.39,0.24	0.650
CYP2C19 UM	-0.04	-0.68,0.61	0.913
CYP2C19 IM : Diabetes	-2.33	-3.41,-1.25	**<0.001**
CYP2C19 PM : Diabetes	1.62	-0.97,4.21	0.221
CYP2C19 RM : Diabetes	-0.76	-1.92,0.40	0.198
CYP2C19 UM : Diabetes	-3.78	-6.28,-1.28	**0.003**
Takes CYP2C19 inhibitor	0.36	0.07,0.65	**0.016**
Sex: Male	0.29	0.02,0.55	**0.032**
Age at recruitment	0.13	0.12,0.15	**<0.001**
Ethnicity: Admix Caucasian	0.02	-0.75,0.80	0.958
Ethnicity: African	1.90	0.58,3.22	**0.005**
Ethnicity: East Asian	0.45	-2.83,3.73	0.788
Ethnicity: Other	0.81	-0.16,1.78	0.100

Ethnicity: South Asian	3.80	2.78,4.81	**<0.001**
Diabetes	7.53	6.55,8.51	**<0.001**
BMI	0.16	0.14,0.19	**<0.001**
Antidiabetics	12.59	11.65,13.52	**<0.001**
Observations		7545	
R^2 / R^2 adjusted		0.470 / 0.468	

Supplementary Table S14. Stratified analysis of people taking citalopram

	Citalopram							
	Diabetes				No diabetes			
Predictors	*N*	*Estimates*	*CI*	*p*	*N*	*Estimates*	*CI*	*p*
CYP2C19 IM	181	-2.42	-4.99, 0.16	0.066	2024	-2.42	-0.29, 0.18	0.635
CYP2C19 PM	19	1.37	-4.81, 7.54	0.664	213	1.37	-0.31, 0.80	0.392
CYP2C19 RM	140	-1.03	-3.82, 1.76	0.470	1742	-1.03	-0.31, 0.17	0.557
CYP2C19 UM	20	-4.07	-10.09, 1.94	0.184	283	-4.07	-0.52, 0.46	0.894
Observations			583		6962			
R^2 / R^2adjusted			0.189 / 0.170		0.127 / 0.125			

Model adjusted by age, ethnicity, sex, taking inhibitors of CYP2D6, taking antidiabetics and BMI

Normal metabolisers of CYP2C19: citalopram diabetes = 223, sertraline diabetes = 74

Supplementary Table S15. Association between CYP2C19 metabolic phenotype and HbA1c within individuals taking sertraline

	Sertraline		
Predictors	*Estimates*	*CI*	*p*
CYP2C19 IM	0.13	-0.49,0.76	0.679

CYP2C19 PM	-0.58	-2.02,0.86	0.429
CYP2C19 RM	-0.17	-0.84,0.50	0.618
CYP2C19 UM	-0.47	-1.82,0.89	0.500
CYP2C19 IM : Diabetes	-0.64	-2.68,1.40	0.539
CYP2C19 PM : Diabetes	-5.84	-11.12,-0.56	**0.030**
CYP2C19 RM : Diabetes	0.17	-1.96,2.30	0.876
CYP2C19 UM : Diabetes	8.52	3.31,13.73	**0.001**
Takes CYP2C19 inhibitor	-0.05	-0.62,0.52	0.860
Sex: Male	0.04	-0.49,0.58	0.876
Age at recruitment	0.13	0.10,0.16	**<0.001**
Ethnicity: Admix Caucasian	-0.37	-1.71,0.97	0.588
Ethnicity: African	2.31	-0.51,5.13	0.108
Ethnicity: East Asian	2.91	-2.51,8.32	0.293
Ethnicity: Other	-0.46	-2.61,1.68	0.671
Ethnicity: South Asian	2.29	0.00,4.57	**0.050**
Diabetes	5.80	4.01,7.60	**<0.001**
BMI	0.18	0.13,0.22	**<0.001**
Antidiabetics	11.57	9.84,13.30	**<0.001**
Observations		1955	
R^2 / R^2 adjusted		0.438 / 0.433	

Supplementary Table S16. Stratified analysis of people taking sertraline

Sertraline								
	**Diabetes**				**No diabetes**			
Predictors	*N*	*Estimates*	*CI*	*p*	*N*	*Estimates*	*CI*	*p*

CYP2C19 IM	54	-2.42	-5.07, 3.85	0.787	533	-2.42	-0.35, 0.59	0.621
CYP2C19 PM	5	1.37	-20.28, 3.49	0.165	62	1.37	-1.66, 0.52	0.306
CYP2C19 RM	47	-1.03	-4.28, 5.10	0.863	418	-1.03	-0.69, 0.33	0.494
CYP2C19 UM	71	-4.07	-3.85, 19.04	0.192	71	-4.07	-1.53, 0.52	0.330
Observations			185			1770		
R^2 / R^2adjusted			0.232 / 0.174			0.133 / 0.127		

Model adjusted by age, ethnicity, sex, taking inhibitors of CYP2D6, taking antidiabetics and BMI

Normal metabolisers of CYP2C19: citalopram diabetes = 223, sertraline diabetes = 74

Supplementary Table S17. Association between CYP2D6 and CYP2C19 metabolic phenotype and HbA1c within Amitriptyline

	**Amitriptyline**		
Predictors	*Estimates*	*CI*	*p*
CYP2D6 IM	0.04	-0.28,0.37	0.789
CYP2D6 PM	0.10	-0.51,0.72	0.740
CYP2C19 IM	-0.10	-0.43,0.23	0.545
CYP2C19 PM	0.01	-0.79,0.81	0.978
CYP2C19 RM	-0.13	-0.49,0.23	0.476
CYP2C19 UM	0.02	-0.66,0.70	0.951
Diabetes : CYP2D6 IM	1.03	0.02,2.03	**0.046**
Diabetes: CYP2D6 PM	-3.41	-5.44,-1.38	**0.001**
CYP2C19 IM : Diabetes	-0.07	-1.12,0.97	0.889
CYP2C19 PM : Diabetes	-1.67	-4.01,0.67	0.163
CYP2C19 RM : Diabetes	0.46	-0.61,1.53	0.401

CYP2C19 UM : Diabetes	0.06	-2.06,2.18	0.955
Takes CYP2D6 inhibitor	-0.28	-0.78,0.23	0.280
Takes CYP2C19 inhibitor	0.37	0.09,0.65	**0.009**
Sex: Male	0.09	-0.20,0.38	0.532
Age at recruitment	0.12	0.10,0.14	**<0.001**
Ethnicity: Admix Caucasian	0.07	-0.84,0.98	0.886
Ethnicity: African	2.57	1.42,3.72	**<0.001**
Ethnicity: East Asian	-1.41	-5.83,3.00	0.531
Ethnicity: Other	1.74	0.66,2.82	**0.002**
Ethnicity: South Asian	2.90	1.81,4.00	**<0.001**
Diabetes	6.68	5.64,7.73	**<0.001**
Antidiabetics	12.36	11.43,13.29	**<0.001**
BMI	0.16	0.13,0.18	**<0.001**
Observations	8191		

Supplementary Table S18. Stratified analysis of people taking amitriptyline

	Amitriptyline							
	Diabetes				No diabetes			
Predictors	*N*	*Estimates*	*CI*	*p*	*N*	*Estimates*	*CI*	*p*
CYP2D6 IM	197	1.18	-0.93, 3.30	0.273	1732	0.04	-0.19, 0.28	0.733
CYP2D6 PM	39	-2.92	-7.23, 1.39	0.184	383	0.10	-0.35, 0.55	0.671
CYP2C19 IM	248	-0.26	-2.46, 1.95	0.819	2235	-0.09	-0.33, 0.15	0.473
CYP2C19 PM	31	-1.43	-6.33, 3.48	0.568	232	0.04	-0.54, 0.62	0.902

CYP2C19 RM	235	0.07	-2.19, 2.32	0.952	1726	-0.12	-0.38, 0.14	0.366
CYP2C19 UM	38	-0.09	-4.55, 4.37	0.969	330	0.05	-0.45, 0.54	0.851
Observations			874			7317		
R^2 / R^2 adjusted			0.158 / 0.142			0.100 / 0.098		

Supplementary Table S19. Association between CYP2D6 and CYP2C19 metabolic phenotype and HbA1c within individuals taking tricyclic antidepressants

	**Tricyclic antidepressants**		
Predictors	*Estimates*	*CI*	*p*
CYP2D6 IM	0.04	-0.26,0.34	0.793
CYP2D6 PM	0.13	-0.46,0.72	0.660
CYP2C19 IM	-0.11	-0.42,0.20	0.495
CYP2C19 PM	-0.07	-0.83,0.69	0.857
CYP2C19 RM	-0.09	-0.43,0.24	0.594
CYP2C19 UM	0.12	-0.52,0.77	0.709
Diabetes : CYP2D6 IM	0.53	-0.43,1.50	0.279
Diabetes: CYP2D6 PM	-3.85	-5.76,-1.95	**<0.001**
CYP2C19 IM : Diabetes	-0.49	-1.48,0.51	0.338
CYP2C19 PM : Diabetes	-1.34	-3.50,0.81	0.222
CYP2C19 RM : Diabetes	0.27	-0.75,1.29	0.604
CYP2C19 UM : Diabetes	-0.52	-2.52,1.49	0.613
Takes CYP2D6 inhibitor	-0.26	-0.73,0.21	0.279
Takes CYP2C19 inhibitor	0.39	0.13,0.66	**0.004**
Sex: Male	0.11	-0.16,0.38	0.428

Age at recruitment	0.12	0.11,0.14	**<0.001**
Ethnicity: Admix Caucasian	0.18	-0.67,1.03	0.686
Ethnicity: African	2.47	1.36,3.57	**<0.001**
Ethnicity: East Asian	0.81	-2.55,4.16	0.638
Ethnicity: Other	2.19	1.16,3.22	**<0.001**
Ethnicity: South Asian	2.55	1.52,3.59	**<0.001**
Diabetes	6.98	5.99,7.97	**<0.001**
BMI	0.16	0.13,0.18	**<0.001**
Antidiabetics	12.45	11.58,13.33	**<0.001**
Observations	9095		
R^2 / R^2 adjusted	0.484 / 0.482		

Supplementary Table S20. Stratified analysis of people taking tricyclic antidepressants

			Tricyclics					
		Diabetes				No diabetes		
Predictors	*N*	*Estimates*	*CI*	*p*	*N*	*Estimates*	*CI*	*p*
CYP2D6 IM	1949	0.73	-1.33, 2.78	0.488	208	0.04	-0.18, 0.26	0.740
CYP2D6 PM	419	-3.30	-7.36, 0.76	0.111	44	0.12	-0.31, 0.54	0.596
CYP2C19 IM	2475	-0.61	-2.73, 1.50	0.570	274	-0.09	-0.32, 0.13	0.414
CYP2C19 PM	252	-1.26	-5.77, 3.25	0.584	37	-0.04	-0.59, 0.52	0.899
CYP2C19 RM	1940	-0.08	-2.24, 2.09	0.945	257	-0.08	-0.33, 0.16	0.501
CYP2C19 UM	362	-0.59	-4.84, 3.66	0.786	42	0.15	-0.32, 0.62	0.526

Observations	955	8140
R^2 / R^2 adjusted	0.162 / 0.147	0.101 / 0.099

Model adjusted by age, ethnicity, sex, taking inhibitors of CYP2D6, taking antidiabetics and BMI, Normal metabolisers of CYP2D6: tricyclics diabetes 703, amitriptyline diabetes = 638, Normal metabolisers of CYP2C19: tricyclics diabetes = 345, amitriptyline = 322

Supplementary Table S21. Antipsychotics regression model. Association between CYP2D6 metabolic phenotype and HbA1c – additional detail

	Antipsychotics		
Predictors	*Estimates*	*CI*	*p*
CYP2D6 IM	-0.02	-0.58,0.53	0.930
CYP2D6 PM	-0.93	-2.01,0.16	0.093
Takes CYP2D6 inhibitor	0.59	-0.43,1.61	0.260
Sex: Male	0.40	-0.07,0.88	0.097
Age at recruitment	0.09	0.06,0.12	**<0.001**
Ethnicity: Admix Caucasian	0.78	-0.67,2.23	0.291
Ethnicity: African	3.81	2.49,5.13	**<0.001**
Ethnicity: East Asian	2.31	-1.21,5.83	0.198
Ethnicity: Other	0.94	-0.70,2.58	0.263
Ethnicity: South Asian	3.72	2.19,5.26	**<0.001**
Diabetes	4.55	3.13,5.97	**<0.001**
BMI	0.19	0.15,0.23	**<0.001**
Antidiabetics	14.18	12.55,15.81	**<0.001**
Observations	2699		
R^2 / R^2 adjusted	0.449 / 0.446		

Supplementary Table S22. Association between CYP2D6 metabolic phenotype and HbA1c among participants taking only antipsychotics, without a co-prescribed antidepressant

	Antipsychotics		
Predictors	*Estimates*	*CI*	*p*
CYP2D6 IM	0.11	-0.56, 0.78	0.754
CYP2D6 PM	-0.78	-2.09, 0.54	0.248
Takes CYP2D6 inhibitor	0.46	-0.88, 1.79	0.502
Sex: Male	0.27	-0.31, 0.86	0.356
Age at recruitment	0.1	0.06, 0.14	**<0.001**
Ethnicity: Admix Caucasian	-0.27	-2.07, 1.53	0.772
Ethnicity: African	3.32	1.89, 4.75	**<0.001**
Ethnicity: East Asian	1.05	-2.99, 5.09	0.61
Ethnicity: Other	1.14	-1.01, 3.29	0.298
Ethnicity: South Asian	4.35	2.62, 6.08	**<0.001**
Diabetes	0.21	0.16, 0.27	**<0.001**
BMI	4.8	2.99, 6.61	**<0.001**
Antidiabetics	11.06	8.92, 13.20	**<0.001**
Observations	1570		
R^2 / R^2 adjusted	0.376 / 0.371		
